# An updated landscape on nanotechnology-based drug delivery, immunotherapy, vaccinations, imaging, and biomarker detections for cancers: recent trends and future directions with clinical success

**DOI:** 10.1186/s11671-023-03913-6

**Published:** 2023-12-19

**Authors:** Pragati Ramesh Kumbhar, Prakash Kumar, Aarti Lasure, Ravichandiran Velayutham, Debabrata Mandal

**Affiliations:** 1 Department of Biotechnology, National Institute of Pharmaceutical Education and Research– Hajipur, Hajipur, 844102 India; 2 National Institute of Pharmaceutical Education and Research– Kolkata, Kolkata, 700054 India

**Keywords:** Nanoparticle, Liposomes, Quantum dots, Nanovaccines, Immunotherapy, Cancer biomarkers, Clinical trial

## Abstract

The recent development of nanotechnology-based formulations improved the diagnostics and therapies for various diseases including cancer where lack of specificity, high cytotoxicity with various side effects, poor biocompatibility, and increasing cases of multi-drug resistance are the major limitations of existing chemotherapy. Nanoparticle-based drug delivery enhances the stability and bioavailability of many drugs, thereby increasing tissue penetration and targeted delivery with improved efficacy against the tumour cells. Easy surface functionalization and encapsulation properties allow various antigens and tumour cell lysates to be delivered in the form of nanovaccines with improved immune response. The nanoparticles (NPs) due to their smaller size and associated optical, physical, and mechanical properties have evolved as biosensors with high sensitivity and specificity for the detection of various markers including nucleic acids, protein/antigens, small metabolites, etc. This review gives, initially, a concise update on drug delivery using different nanoscale platforms like liposomes, dendrimers, polymeric & various metallic NPs, hydrogels, microneedles, nanofibres, nanoemulsions, etc. Drug delivery with recent technologies like quantum dots (QDs), carbon nanotubes (CNTs), protein, and upconverting NPs was updated, thereafter. We also summarized the recent progress in vaccination strategy, immunotherapy involving immune checkpoint inhibitors, and biomarker detection for various cancers based on nanoplatforms. At last, we gave a detailed picture of the current nanomedicines in clinical trials and their possible success along with the existing approved ones. In short, this review provides an updated complete landscape of applications of wide NP-based drug delivery, vaccinations, immunotherapy, biomarker detection & imaging for various cancers with a predicted future of nanomedicines that are in clinical trials.

**Graphical abstract:**

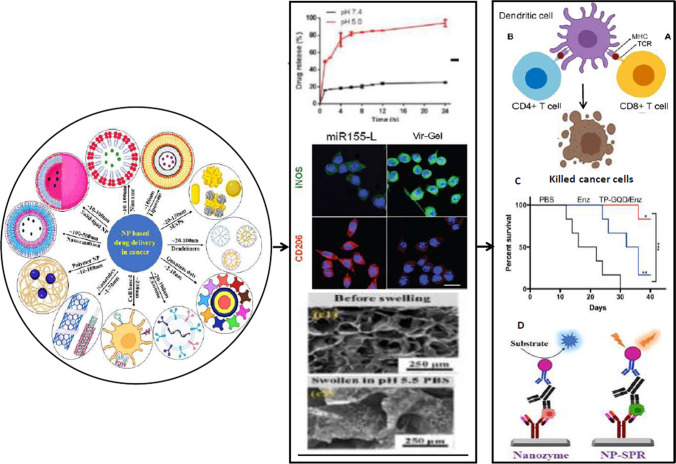

## Introduction

The Greek physician Hippocrates (460–370 BC) was the first person to coin the terms ‘carcinos’ and ‘carcinoma’ to define non-ulcer-forming and ulcer-forming tumours. The Roman physician, Celsus (25 BC-50 AD), translated the Greek term carcinos into cancer which means crab in Latin. Further, the term oncology came from Galen (130–200 AD, Greek physician) who used the term ‘oncos’ (which means swelling in Greek) to describe tumours. In the modern era, cancer is a group of diseases which is characterized by abnormal cell growth which can infiltrate or spread quickly to other parts of the body [1]. However, benign tumours do not spread. A lump, a persistent cough, unexplained weight loss, unusual bleeding, and a change in bowel movements are all possible indications and symptoms of various forms of cancer. Although these signs/ symptoms may suggest cancer, they could coincide with other diseases, which are not life-threatening, and thereby detection of cancer at early stages is mostly overlooked. Humans are affected by about 100 different types of cancer. Among these cancers, leukaemia which is a group of blood cancers and cancers affecting different organs of the body like breast, prostate, lung cancer, etc., is most common and, therefore, chemotherapy and anticancer drug delivery against these cancers are always in high demand. Currently, significant progress has been made in developing nanotechnology-based anticancer drug delivery targeted against the malignant cells to kill and/or reduce the proliferation of these cells without affecting the healthy cells [2].

In 2018, the number of new cancer cases crossed > 18 millions with > 9 million deaths by different forms of cancer across the globe [3]. In 2020, the number of deaths from cancers was > 10 million where lung, colon & rectum, and liver cancers contributed to 1.8, 0.9, and 0.83 million deaths, respectively [4]. Breast cancer is still the most common with nearly 2.25 million cases. Cervical cancer is the most widespread (in 23 countries) and cancer cases for children are ~ 0.4 million. According to the Global Cancer Observatory (GCO), nearly 30–32 million cancer patients will die from cancer each year by 2030 [5] even though most of the R&D-based research is focused on anticancer drug delivery around the world. Apart from the high mortality of cancer, the economic burden of treatment requirements on families of cancer patients and society is enormous and growing regularly. The emergence of drug resistance against standard chemotherapy adds a new dimension to this existing problem. Therefore, efforts on cancer prevention, diagnosis, and treatment with reduced cost and toxicity against resistance cases of cancer are of great importance. Cancer is a multistep process that occurs over time with acquired genetic mutations. The cancer cells become abnormal after gaining additional capacities such as the ability to divide in an uncontrolled manner. Moreover when the cells continue to divide, they affect neighbouring normal cells and as a result, demising the organ’s function [6]. Nanomaterials have a wide range of applications, ranging from drug/peptide/small molecule delivery, siRNA delivery for gene-based targeting, biomedical imaging, etc. This is due to their lower size range (1–100 nm), increased surface area allowing a vast range of molecules to be conjugated and/or functionalized, and unique mechanical, optical, and magnetic properties associated with different synthesis procedures of these NPs. Nanoplatforms such as liposomes, lipid NPs, dendrimers, micelles, hydrogels. Microneedles, nanofibres, and metallic NPs can help in cancer diagnosis and treatment [7] due to fast & targeted delivery, increased drug load at the target site, and reduced toxicity.

In this review, we intended to provide a broad detail of NP-based applications for various cancers which include drug delivery, vaccinations, immunotherapy, and biomarker detection & imaging with NPs. Before that, we have described the brief history of cancer chemotherapy, monoclonal antibody-based therapy, chimeric antigen receptor (CAR)-based modern T cell therapy, and their current limitations to justify the progress made in NP-based therapy. In the drug delivery part, there are 16 different NP-based delivery methods were discussed among which liposomal, polymeric, hydrogel, and metallic NP-based delivery are described in more detail since they are the more common. In the drug delivery sections, we have also included some of the most modern-day technologies like microneedles, nanofibres, exosomes, graphene and carbon quantum dots, carbon nanotubes, and upconverting NPs with the latest examples of their applications. In the immunotherapy section we have outlined the progress and challenges made in immune checkpoint inhibitors and the related NP-based applications. The chapter on clinical trials gives an overview of different NP-based anticancer drug delivery which is different phases of trials along with details of approved ones. We have also included a chapter describing very common challenges in nanomedicine like regulatory barriers, large-scale production, toxicity, failures of nanomedicine drugs, and problems associated with protein corona in NP-based delivery.

## Limitations of conventional chemotherapy and current treatments for cancers

Chemotherapy, the most widely used cancer treatment method, was first introduced in the year 1942 by Drs. Gilman, Goodman, and Gustaf Lindskogas 0.1 mg/kg^−1^ of synthetic “lymphocidal chemical” to treat a person who was a victim of mustard gas poisoning during WWII [8]. It was based on toxicological safety against rabbits for the same chemical. The chemotherapy showed early signs of recovery with reduced pain but failed to save the patient in the longer run. Discovery of 5-fluorouracil (5-FA), methotrexate, and cisplatin during the years 1956, 1958, and 1965, respectively, gave a breakthrough in the chemotherapy of cancers. Chemotherapy works through several different mechanisms although its major function includes the faster killing of vigorously growing cells, including tumour and normal cells thereby associated, invariably, with serious side effects including hair loss, bone marrow suppression, gastrointestinal reactions, etc. [9]. These side effects are almost unavoidable since cells associated with hair follicles, GI tract, and bone marrow are also rapidly growing cells in human bodies, and the anticancer agents cannot distinguish them from fast-growing tumour cells as targets. Therefore, development of the anti-cancer drugs that precisely target tumour cells, instead of normal cells, is of utmost interest. Although targeted therapy has made great progress, there are still many unavoidable and severe adverse effects and the emergence of drug resistance has always been a growing concern.

The World Health Organization (WHO) has graded the side effects of cancers from 0 to 4 based on the severity. Side effects of chemotherapy include immediate symptoms of mild toxicity and late signs of chronic toxicity (4,5). According to the WHO classification their intensity can be classified as grade 1 (mild), grade 2 (moderate), grade 3 (severe) (grade 3), or grade 4 (life-threatening or disabling). Immediate effects of anti-cancer treatment can be observed on skin and hair, the gastrointestinal tract, the kidneys, blood, and bone marrow, thereby damaging immune functions. Grade 3 and 4 neurotoxicity can be associated with somnolence, paralysis, paresthesia, spasms, ataxia, and coma. In the late 1970s, due to a lack of antiemetic drugs the chemotherapy of bleomycin, vinblastine, and cisplatin was tolerated with vomiting of even > 10 times per day as a common side effect [9]. Approved cytostatic drugs like alkylating agents, antibiotics, alkaloids, and antimetabolites were found to be associated with relatively low tumour specificity and high toxicity. The 5-year survival, from only chemotherapy, was estimated to be < 3% in the USA and Australia based on a clinical trial study that includes > 150,000 and > 72,000 cancer patients undergoing different chemotherapy in these two countries, respectively [10]. Based on the WHO report in 2014, it was observed that in Europe lung cancer had the highest rate of mortality (20%) and stomach cancer (6.1%) had the lowest [11, 12]. The mortality rate for colorectal cancer was > 12%. Even though significant progress in anticancer treatment was achieved in the later years, the American Cancer Society reported that during the 2018–2019 period, the reduction of cancer mortality was only 1.7% from the previous year’s [13]. Meta-analyses of the toxicity of novel drugs, between 2008 and 2010 approved by the Food and Drug Administration (FDA) showed novel drugs were associated with a significantly higher risk of harm than the standard therapy like cisplatin or 5-FA [14]. These data indicate the urgent need for a better delivery system than standard chemotherapy.

The discovery of antibody–drug conjugates (ADCs) and T cell-based therapy with chimeric antigen receptors (CARs) has revolutionized modern-day cancer treatment. ADCs are a promising delivery system comprising a carrier (generally a monoclonal antibody), linker, and cytotoxic payload specific for tumour cells [15]. The carrier binds to a target molecule that is overexpressed in tumour cells, whereas the linker conjugates the payload to the constant region of the heavy chain of the antibody. Due to the specific binding of a target with a receptor in the cancer cells the payload can deliver the therapeutic effect specifically against cancer cells. Since the first ADC, mylotarg (gemtuzumab ozogamicin), which was approved by the FDA in 2000 [16], 14 ADCs have been approved for the therapy of various cancers. Despite the rapid development of ADCs, there are severe limitations for their use as carriers, such as the high cost of production for monoclonal antibodies& possible loss of function due to linker chemistry, low penetration ability, and potentially life-threatening side effects due to off-target binding [16, 17]. Chimeric antigen receptors (CARs) are receptor proteins that have been genetically engineered to give T cells the novel ability to target a specific antigen. The receptors are called chimeric because they have dual functions of antigen-binding and T-cell activation from a single receptor. CAR T cells can be derived autologously (T cell from a patient’s blood) or allogenically (T cell from a donor’s blood) which is engineered using recombinant DNA technology to express a specific CAR (CD3ζ, CD28, etc.), which enables them to target an antigen that is abundant/overexpressed on the surface of tumours. Since 2017, the FDA approved six CAR T-cell therapies for the treatment of blood cancers, including lymphomas, and, most recently, multiple myeloma. Despite the progress, they allow long-term survival in very few treated patients. The expenses for CAR T-cell therapy are more than $450,000 in some cases, and there are serious side effects like cytokine release syndrome, neurological toxicity, etc. [18]. Therefore, even though chemotherapy is an old method, its use using a nanotechnology-based platform may have a better future, at least, for the next few years. This is due to the high cost-to-benefit ratio for ADCs and CAR-T cell-based treatment and the lack of enough toxicological data and related side effects due to the limited use of these advanced technologies to date.

## A cancer-treatment delivery system based on nanotechnology

**Fig. 1:**
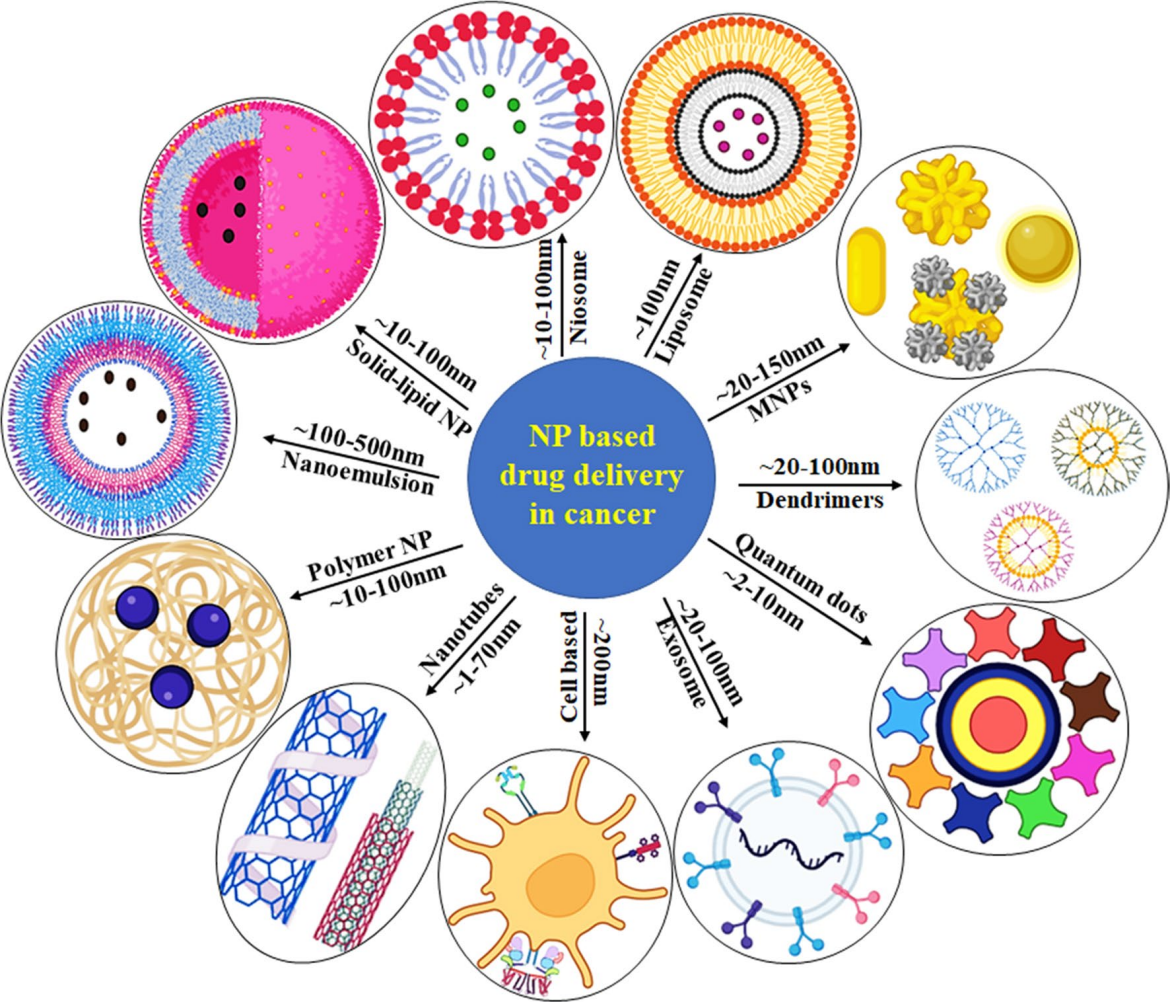
A summary of different nanoparticle-based drug delivery against cancer with their size, shape, and morphological features

Nanotechnology has a better potential to improve cancer diagnostics than standard chemotherapy. One of the most common applications of NPs and nanoformulations is to improve drug delivery. Liposomes, dendrimers, nanoshells, micelles, polymeric nanocarriers, and metallic NPs, which are some of the most common nanotechnology frameworks for cancer therapy, are discussed along with recent developments with QDs, CNTs, upconverting, and protein NPs [19]. Apart from that hydrogel, microneedle, and nanofiber-based drug delivery was discussed. In Fig. 1, different NP-based delivery systems which are used for cancers are summarized with size, shape, and morphological features.

## Liposome

The United States Food and Drug Administration (USFDA) has approved the first nanomedicine for clinical use which is a liposomal formulation (Doxil®-doxorubicin hydrochloride PEGylated liposomal formulation in 1995, and produced by Sequus Pharmaceuticals, USA) [20]. It is noteworthy that liposome-based formulations are most common in drug delivery and are also approved clinically for other diseases like AmBisome for parasitic/fungal infections. Liposomes, which create lipid bilayers through hydrophobic contact, are regarded as effective platforms for the delivery of hydrophobic and hydrophilic molecules. Liposomes, which are highly biocompatible, are generally 200 nm or less in size, referred to as nanoliposomes [21]. Liposomes are the simplest form of nanovectors, consisting of lipids encircling a water core where a closed spherical vesicle made up of a lipid bilayer that encases an aqueous phase for drug storage [22]. Liposomes are generally bigger than the standard NPs [23]. The liposomal co-delivery of omacetaxine mepesuccinate (OMT) and DOX showed synergism [24] against cervical carcinoma HeLa cells. Further, this combination in a molar ratio of 4:1 reduced the tumour growths, in vivo, by 98.6% and 97.3% relative to the untreated control on days 15 and 25 after the cessation of treatment, respectively. Compared to conventional chemotherapeutics with cisplatin and paclitaxel, novel liposomal NP-modified valeric acid effectively retarded the growth of human non-small cell lung cancer (NSCLC) cell lines to a greater extent and also blocked further progression of tumour tissues in vivo with lower cytotoxicity towards normal lung cell lines [25]. The mechanistic pathway of valeric acid is involved with downregulation of Signal transducer and activator of transcription-3 (STAT3)/cyclin D1 pathway which is directly related to tumour cell proliferation. It is indeed a very rare study where a small chain fatty acid (SCFA) was shown to be more effective than cisplatin and paclitaxel in a liposomal formulation although there are reports of SCFAs, composed of 7 metabolites from gut microbiota *E. coli KUB-36* strain, having strong anticancer effect on breast cancer cells [26]. The topoisomerase I inhibitor 7-ethyl-10-hydroxycamptothecin (SN38) as a model drug and its conjugate with cholesterol (Chol-SN38@LP) as a prodrug with cleavable esterase activity showed preferential accumulation of therapeutic payloads in tumour lesions [22]. Moreover, this liposomal prodrug formulation showed improved drug tolerability with alleviated bloody diarrhoea and liver damage in multiple mice models compared to the standard SN38 clinical counterpart of irinotecan. Oxaliplatin (OXP) is the typical treatment for colorectal cancer, whereas hesperidin (HSP) is a natural anti-cancer flavonoid. Co-delivery of OXP with HSP with cationic Okra gum (COG)-coated nanoliposomes with particle sizes of 145–175 nm showed significantly improved efficacy against colorectal cancer cells than normal drug combinations [27]. Further, combining liposomal delivery with ultrasound treatment was found to be effective in the delivery of payload with reduced toxicity in anticancer studies [28]. Niosomes are slightly different from liposomes as the bilayers are formed by non-ionic surfactants. The oxazaphosphorine family of alkylating drugs, Ifosfamide (IFO), when loaded with niosomes, showed potent activity with IC_50_ of < 0.2 µg/ml against neuroblastoma SH-SY5Y cells [29] along with improved biochemical parameters against rat model of cancer with intravenous delivery of 0.2 mg/Kg of body weight. For liposomes, the stimuli-responsive drug delivery, where the stimuli can be pH-, redox-, enzyme-, light-, thermo-, and magneto-sensitive, is another advantage. For example, the pH-sensitive liposomes release the drug in response to a mildly acidic pH, thereby allowing drug endosomal escape with improved stability [30]. The liposomal structure known as liposomes in (composed of phospholipids) was solved by Dr. Alec Bangham, and at first, their group coined the term liposomology in the 1960s to describe the lipid-based drug delivery system [31]. The biggest advantage of the liposomes (which range from 30 nm to the micrometre scale with an 4–5-nm-thick outer-layer) is that they can protect the encapsulated drugs from harsh physiological degradation, thereby extending the half-life of the drug with sustained release, biocompatibility, and safety. The only disadvantage for lipid-based delivery compared to other delivery systems is the high cost associated with some of the lipids/phospholipids. For example, lipid-based formulation AmBisome (which is used for fungal and parasitic diseases), is so expensive that alternative delivery options and clinical trials were done to check the reduced cost and safety of the associated drug [32, 33].

## Polymeric nanoparticles

**Fig. 2:**
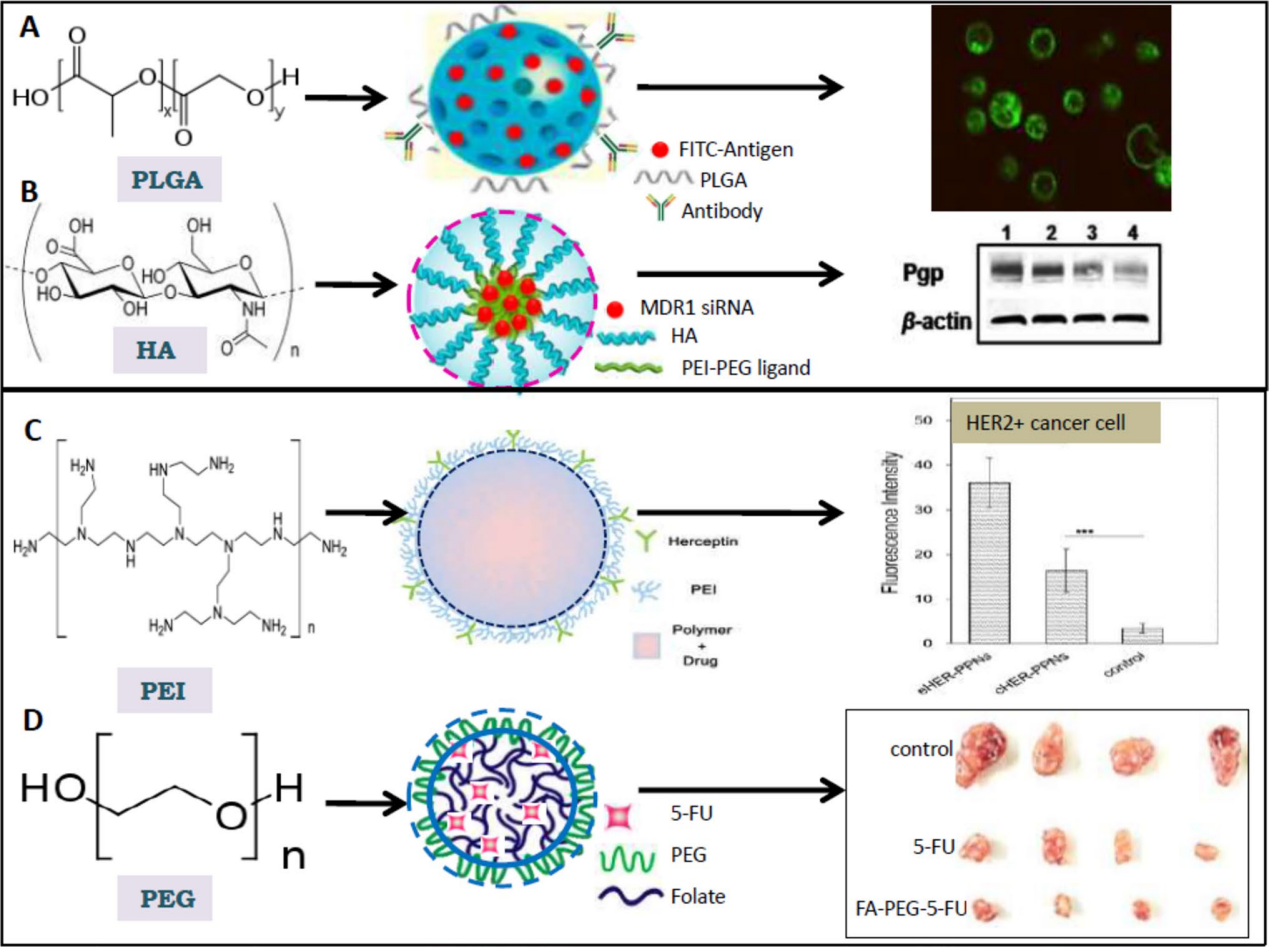
Different polymeric NPs for anticancer drug delivery. **A** PLGA NPs carrying FITC antigen targeted for DCs are localized to membranes of DCs with better antigen presentation. **B** HA-based NPs carrying increasing doses of MDR1 siRNA decrease the P-glycoprotein (Pgp) expression in MDR strains of ovarian cancer. **C** Herceptin-bearing PEI/PLGA NPs (eHER-PPNs) deliver more paclitaxel in Her2^+^ cancer cells as measured by increased fluorescence intensity. **D** Folate-conjugated prodrug of PEG (FA-PEG5-FU) decreases the tumour size with increasing days in mice much better than 5-FU. The **A** and **B** were reproduced/adapted with permission from [Elsevier] for reference [38], and from [springer Nature] for reference [42], all rights reserved. **C** and **D** were adapted from references [48] and [49], for which copyright permission is not required

A natural and synthetic polymer is used to make polymeric nanocarriers. Polymeric nanocarriers, also known as nanospheres (matrix type of structure) or nanocapsules (vesicular system), are made by binding a copolymer to a polymer matrix and are one of the well-studied drug delivery nanoplatforms within a size range 10–1000 nm. Polymeric NPs (PNPs) do not extend their hydrophobic tail like polymeric micelles in the surrounding aqueous layer. Biodegradable polymers like poly(ethylene glycol) (PEG), poly(ethyleneimine) (PEI), poly(D,l-lactide) (PLA), poly(D,L-glycolide) (PLG), co-polymer poly(lactide-co-glycolide) (PLGA), polyalkylcyanoacrylates, poly-Ɛ-caprolactone are biocompatible and generally recognized as safe (GRAS) by USFDA for drug delivery [34]. Moreover, degradation products of these polymers are eliminated from the body in the simplest form of CO_2_ and water. Toll-like receptor-7 (TLR-7) agonist, imiquimod, embedded in PLGA NPs is functionalized with the cancer cell membrane to expose the membrane proteins as antigens. For higher uptake of NPs in antigen-presenting cells, like macrophage or dendritic cells, mannose was crafted and modified on the surface of NPs. These nanoengineered PLGA NPs showed significant inhibition of tumour growth in the animal model [35]. PLGA NPs (~ 270 nm with a surface charge of ~ 42 mV) loaded with brigatinib, an epidermal growth factor receptor (EGFR) inhibitor, showed the IC_50_ of 5.25 μg/ml against lung carcinoma cell line A549 [36]. PLGA-mediated immune therapy was also developed for cancer treatment. In cancer immuno-therapy, CD8 +-associated antigen-specific T lymphocyte response is important to kill the cancer cells by activating the cytotoxic T lymphocyte (CTL) response from dendritic cells (DCs) [37]. In an earlier study, the human DC-targeted antibody (hD1) was used to target the C-type lectin DC-SIGN onto a lipid-PLGA-PEG layer carrying FITC-tagged antigens (Tetanus Toxoid and BSA). The NP (200 nm) or microparticles (2000 nm) carrying these antigens show increased internalization against DCs as observed by fluorescence microscopy [38]. The PLGA NP-based delivery allowed slow but sustained degradation of antigen (38% antigen degradation in 6 days) with a T-cell response that is effective with a 100-fold lower concentration of the antigen than the non-targeted delivery system. The PLA-based copolymeric nanocarrier (NAV/DCB NPs) for the co-delivery of navitoclax (a Bcl-2 inhibitor) and decitabine (a nucleic acid synthesis inhibitor) showed potent in vitro synergistic cytotoxicity against both acute myeloid leukaemia (AML) and breast cancer cell lines with potent tumour growth inhibition on the AML xenografts also [39]. The lipid-polymer hybrid nanoparticles (LPNP) encapsulated with DOX and alpha-tocopherol succinate (TS) with PLGA as the polymer (LPNP-TS-DOX) showed increased cellular uptake for the 4T1breast cancer cell line compared to free DOX with higher in vivo antitumour activity [40]. The cellular uptake, cytotoxicity, and cell migration studies showed that the formulation LPNP-TS-DOX is more effective compared to free DOX against 4T1 (BALB/c mouse strain) cells than human breast cancer cell lines (MCF-7 and MDA-MB-231) in vitro. Breast cancer is associated with overexpression of transmembrane tyrosine kinase HER2 and, therefore, antibodies targeting the HER2 receptor but conjugated with polymeric NPs have been widely used [41]. In Table 1 liposomal and different polymeric NPs for anticancer drug delivery with possible targets were summarized. In Fig. 2, the liposomal and different polymeric NP-based anticancer drug delivery with efficacy and target was shown from the published data with permissions.

**Table 1:** Liposomal and different polymeric NPs for anticancer drug delivery with possible targets on tumour cells and tumour-bearing mice

Type of NP	Composition/Drugs	Study model	Type cancer	Possible molecular mechanism	Refs.
Liposomes	OMT-loaded liposomes,	HeLa cells, Mice model	Cervix adenocarcinoma	Decrease in Mcl-1 levels	[24]
	Valeric acid-loaded liposomes	H1299	Non-small cell lung cancer	Release STAT3/Cyclin D1 pathway	[25]
	Chol-SN38@LP	A549	Lung cancer	Inhibition of topoisomerase I	[27]
	Cationic Okra gum-coated nanoliposomes	HT-29	Colorectal cancers	Catalase (CAT) target	[28]
Polymeric NPs	Poly(d,l-lactide-co-glycolide)	B16-OVA cancer cells	B16-OVA melanoma tumours	Agonist against toll-like receptor 7 (TLR-7)	[35]
	PLA-based copolymeric nanocarrier (NAV/DCB NPs)	MCF-7	Breast cancer	Bcl-2 inhibitor and nucleic acid synthesis inhibitor	[39]
	PLGA-TPGS-Based Hybrid Nanoparticles Loaded with Doxorubicin	4T1, MCF-7, and MDA-MB-231 cell lines	Breast cancer	Targeting the HER2 receptor	[41]
	HA-PEI/HA-PEG and loaded with MDR1 siRNA	OVCAR8TR cells	Ovarian cancer	Binding with CD44 receptor	[42]
	HA-CH-NP/siRNA	A2780, HeyA8, and SKOV3	Ovarian cancer	Targeting the CD44 receptor	[43]
	HA-Lip-HNK	4T1 cells	Breast cancer	Endoplasmic reticulum and mitochondrial dysfunction	[44]
	HA-IR-Pyr	HeLa cell lines	Cervical cancer	ROS generation	[45]
	PEI-coated PLGA/lipid nanoparticles	A549, HepG2, and MCF7 cancer cells	Lung cancer, Hepatocarcinoma, Breast cancer	Binding to microtubules and inhibiting the depolymerization of microtubule	[46]
	HA-modified stearic acid SA)-loaded PEI nanocarrier	OVCAR-3	Ovarian cancer	Targeting folate receptors	[47]
	Herceptin conjugated with PEI/PLGA NPs	BT474 cells MCF7 cells	Breast cancer	Targeting HER2 receptor	[48]
	FA-PEG-5-FU	HeLa cancer cells, Mice model	Cervical cancer	Target folate receptors	[49]

Hyaluronic acid (HA) is a linear anionic polymer, with high biocompatibility, biodegradability, and non-immunogenicity. The crystal structure data at 1.25 Å resolutions confirming the specificity of HA binding with CD44 receptor (which is highly expressed in many cancers) have revolutionized HA-based payload delivery for cancer therapy [50]. To overcome multidrug-resistant (MDR) ovarian cancer the NP system composed of HA-PEI/HA-PEG and loaded with MDR1 siRNA was delivered against established paclitaxel-resistant tumours (OVAR8TR) xenografted nude mice [42]. This delivery downregulates P-glycoprotein (Pgp) suppression within 24 h with a threefold reduction in tumour volume after 30 days with no increased body weight or toxicity against the mice. PLXDC1 (angiogenic marker gene) siRNA-incorporated chitosan NP coated with HA (HA-CH-NP/siRNA, size of 200 nm) reduced the tumour growth in epithelial ovarian cancer with ~ 2.1-fold increased binding of NP on cancer cells with significant reduction of PLXDC1 expression [43]. A cationic liposomal NP composed of HA and honokiol (a lipid-soluble biphenol known to interact with membrane proteins and reduce inflammation) was investigated against breast cancer (4T1 cells) in vitro followed by 3D model spheroids of breast cancer. The HA-Lip-HNK exhibited higher tumour accumulation, with a tumour growth inhibition rate of 60% where increased CD44-HA interaction was confirmed by confocal microscopy [44]. Synthesized mitochondria targeting water-soluble indocyanine dye with a positively charged pyridinium moiety and HA (HA-IR-Pyr) form micelles which were used for photodynamic therapy (PDT) against squamous carcinoma-based skin cell line [45] with increased tumour accumulation. These data suggest that the mere presence of HA in NP is enough for entering various cancer cells through the CD44 receptor. Polyethyleneimine (PEI), a cationic polymer (used as a transfection agent and gene therapy), contains repeating units of primary amino groups and ethylene. Four kinds of PEIs (0.8 kDa-, 2 kDa-, and 25 kDa-branched PEIs, and 25 kDa-linear PEI) coated with PLGA/lipid nanoparticles (PPLs) were prepared (size range 135–535 nm, zeta potential 13.5- 45.4 mV) to check anticancer efficacy against various cancer cell lines [46]. It was observed that 25 kDa-branched PEI-coated PLGA/lipid NPs (25 k-bPPLs) had a higher anticancer activity with excellent colloidal stability in pH 7.4 PBS. This study provided evidence that 25 kDa branched PEI is optimal for anticancer drug delivery. A double-layered HA-coated PEI-g-stearic acid (PgS) was delivered with a chiral drug, (−)-gossypol, for cancer therapy with excellent in vivo efficacy. Here opposite charges of HA and PEI allowed them to combine along with the drug gossypol which is a polyphenolic compound derived from the cotton plant (genus Gossypium) and known to inhibit dehydrogenase enzymes [51]. HA-modified stearic acid (SA)-loaded PEI nanocarrier(~ 189 nm) was co-delivered with curcumin and paclitaxel to exert synergistic anti-ovarian cancer effects on multi-drug resistant variant (SKOV3-TR30) in vitro, with good efficacy against ovarian tumour-bearing nude mice [47]. In another study, Herceptin was electrostatically conjugated on the surface of PEI/PLGA NPs (eHER-PPNs, size ~ 280 nm, zeta potential ~ 1.5 mV) to target HER2-positive BT474 cells and HER2-negative MCF7 cells [48]. The NP eHER-PPNs displayed higher cellular uptake efficiency and anticancer effect than chemically conjugated Herceptin-bearing PEI/PLGA NPs (cHER-PPNs). Apart from PLGA, PEI, and HA polymer-based drug delivery, PEGylated drug delivery is very common for anticancer therapy. Further PEGylation of protein and monoclonal antibody is amply used for anticancer studies. A PEGylated version of interferon-α2b (Sylatron™), which was approved by the FDA for the treatment of melanoma in the year 2011, displayed a half-life of 27–37 h with tenfold lower clearance in comparison with the native form [52]. PEGylated formulations of different low molecular weight anti-cancer drugs which are topoisomerase I inhibitors (irinotecan, topotecan, SN38, exetecan, etc.) are in different phases of the clinical trial for the treatment of many solid tumours [53]. Among different polymers PEGylated NPS top the list of FDA-approved nanomedicine for cancer therapy. In fact, DOXIL (PEGylated liposomal Doxorubicin, FDA approval -1995) is the first FDA-approved nano-drug in the market. DOXIL is enclosed in an 80–90 nm size uni-lamellar liposome coated with PEG providing a long circulation time of about 60–90 h for doses in the range of 35–70 mg/m^2^ in patients with solid tumours [54]. PEGylated delivery allows a wide temperature-sensitive drug delivery. For example, ThermoDox, a temperature-sensitive doxorubicin-loaded PEGylated liposome (DPPC), releases encapsulated doxorubicin at elevated tissue temperature of 41.5 °C where the concentration of the drug > 25 times more than the intravenously treated doxorubicin [55]. To employ folate receptor-mediated drug delivery against cancer cells folic acid (FA), bis-amine polyethylene glycol (PEG), and an anticancer drug, 5-fluorouracil (5-FU) were conjugated to create a prodrug FA-PEG-5-FU. Folate conjugate showed steady drug release for 10 days with improved uptake in the HeLa cancer cells than Vero cells. Further, FA-PEG-5-FU-treated mice group showed ~ 3.2-fold decreased tumour volume after 20 days of treatment [49]. Compared to lipid-based delivery the advantage of polymeric delivery is its low cost and higher biocompatibility. Further, due to its polymeric nature the size of the polymer with entrapped drugs can be easily controlled by simpler polymerization reactions. The easy bio-degradability of FDA-approved polymers (PLGA, PGA, PLA etc.), which is converted to CO_2_ and water inside the body, has encouraged the development of many more delivery systems using these polymers owing to their safety. The major problem of polymeric NPs is the uncontrolled polymerization reaction which may create a widely variable size of NPs and aggregation causing problems with drug loading and release.

## Metallic nanoparticles

Metal NPs are submicron-scale entities consisting of pure metals or their compounds (for example; gold, silver, zinc, cerium, iron, silica, etc.) in the form of oxides, hydroxides, sulphides, phosphates, fluorides, chlorides, etc. [56]. The method of metallic NP production has a significant influence on the size and shape of tiny particles. Different physical, chemical, and biological methods are used to synthesize/functionalize the metallic NPs where the biological/chemical reagents act as reducing and/or capping agents on the surface of NPs [57, 58]. Metallic NPs have been studied in research laboratories more often than any other NPs. This is due to the following reasons—(a) faster and easy synthesis with scalability, (b) green synthesis using various plant extracts/metabolites/natural products, and (c) easy functionalization and delivery of the drug since it is coated on the surface of metal NPs and not entrapped like liposomal and polymeric delivery systems. There are two major problems associated with metallic NPs. Due to their high surface-to-charge ratio, they associate with a high amount of plasma proteins to form a bigger size of NP having protein corona which effectively masks the delivery and activity of the drug on the target site. Another problem of metallic NP-based delivery is the deposition and accumulation of metals on tissues/organs with unknown metabolic fate leading to long-term toxicity. This event further raises the concern about crossing the blood–brain barrier (BBB) of these metallic NPs with harmful side effects.

### Gold nanoparticles

Gold nanoparticles (GNPs) are synthesized biologically or chemically and are one of the most studied NPs for drug delivery. Gold nanospheres, nanorods, nanoshells, and nanocages, for example, are used extensively for imaging, cancer therapy, and drug delivery [6]. Both active and passive targeting were observed for GNPs where the passive targeting mechanism is guided by the gathering of GNPs to enhance imaging due to the enhanced permeability and retention (EPR) effect on tumour tissues. On the other hand, active targeting is achieved by delivering GNPs with tumour-specific targeted receptors/antigens for example GNP-conjugated EGFR monoclonal antibodies for binding with over-expressed receptors on tumour cells [59]. The polyethyleneimine (PEI)-coated cationic GNPs (AuPEI) shifted the immunological tumour microenvironment (TME) in the mouse breast MET-1 tumour model, in vivo, toward an M2 phenotype indicating immunosuppressive profile as determined by the increased PD-1^+^(programmed death ligand)-positive lymphocytes [60]. The increase of PD-1^+^ CD8^+^ T cell population from 33.25 to 63.22% in presence of 2.5 µg/mlAuPEIs, in vivo, may increase the therapeutic efficacy of additional anti-PD-1 or anti-PD-L1 antibodies with better anticancer effect. GNPs covered with DOX and DNA were found to be equally effective in the annihilation of both prostate and liver cancer cells, in vitro [61]. Here DNA was used to induce various conformational changes in the GNP-DOX complex, thereby increasing its efficacy. A glucose-modified dendrimer-entrapped GNPs (Au-DENPs) were prepared and labelled with radionuclide ^68^ Ga and incorporated with CpG (cytosine phosphate guanine, a potent adjuvant) oligonucleotide for positron emission tomography (PET)/computed tomography (CT) dual-mode imaging and immunotherapy of tumours. The developed 2-amino-2-deoxy-D-glucose (DG)-modified GNPs (DG-Au DENPs/CpG) showed a more sensitive imaging effect and better inhibition effect on tumours providing both, theranostic & therapeutic mode of the synthesized GNPs [62]. Ghassan M. Sulaiman et al., 2020 used hesperidin (a flavonoid)-functionalized GNPs for the treatment of the human breast epithelial cell lines. Efficacy was ~ twofold higher for GNPs with very less toxicity. Further, the poor water solubility of hesperidin was improved after functionalization with GNPs [63]. *Taxus baccata* extract-based green synthesis of GNPs (~ 20 nm) showed broad-range anticancer efficacy, in vitro, against MCF-7, Hela, and Caov-4 which are breast, cervical, and ovarian cancer cell lines, respectively [64]. Apart from chemotherapy, GNPs due to their unique optical properties allow radio-therapy and PDT (photodynamic therapy). The CeO_2_-coated gold nanorods were placed onto the surface of the temperature-sensitive polymer followed by layering with the photosensitizer Ce6. Under infrared light in the 660–808 nm range the gold nanorods achieve photothermal conversion inducing Ce6 to produce singlet oxygen, thereby effectively killing all cancer cells in PDT [65]. Gold nanorods are GNPs that have the shape of a rod and follows the non-radiative oscillation decay [66]. Nonradiative decay converts the light energy to heat energy after treatment with near-infrared (NIR) light at 650–1100 nm showing a strong photothermal phenomenon [67]. Nanorods functionalized with Arginyl-Glycyl-Aspartate (RGD) tripeptide, were synthesized by the seedless growth method. The size was ~ 25 nm × 6 nm (length × width), and it inhibited the HeLa cell line growth by altering the cell junction and actin network by the photothermal mechanism of action [68]. Anticancer applications of various metallic NPs including GNP, SNP, INP, ZnO, and Silica NPs are summarized in Table 2.

**Table 2:** Metallic NPs-based drug delivery against different cancer with the possible mechanism of actions and targets

Type of NP	Composition/drugs	Study model	Type cancer	Possible molecular mechanism	Refs.
GNP	GNP-conjugated EGFR monoclonal antibodies	Mice	Pancreatic	Epidermal growth factor receptor-mediated specific delivery to cancer cells	[59]
	GNP-polyethyleneimine	Raw264.7/Mice model	Triple-negative breast	NF-α, CCL5, IL-10, and PD-1 immunomodulation-based therapy	[60]
	Au@16-Ph-16 cationic nanoparticles and DNA-Doxo complexes	SNU-387 and LNCaP cancer cell line	Prostate and hepatocarcinoma	Drug compatibility and internalization were high. ROS-mediated cell killing	[69]
	Dendrimer-entrapped gold nanoparticles (Au DENPs) labelled with radionuclide 68 Ga and CpG	C57BL/6 female mice	Subcutaneous B16 tumour	CD8 + T cells activate the Fas–Fas ligand signalling to clear the cancer cells	[62]
	Hesperidin-GNP	MDA-MB231/Mice	Breast	Apoptosis and inhibition of IL-1β, IL-6, and TNF cytokines	[63]
	Taxus baccata extracts-GNP	MCF-7, HeLa, and Caov-4 cell lines	Breast, cervical, and ovarian cancer	Caspase-independent death program	[64]
	CeO2-coated gold nanorods	HepG2 cell line	Anaerobic tumour microenvironment	Near-infrared (NIR)-808-nm-mediated photodynamic therapy	[65]
	Au-nanorods functionalized with RGD tripeptide	HeLa cell line	Breast	It killed the cancer cells by altering the cell junction and actin network by the PDT mechanism	[68]
SNP	*Pinus roxburghii* extract-SNPs	PC-3 cell line	Prostate	Apoptosis-mediated DNA damage and caspase-activated cell killing of prostate cancer cells	[69]
	*Phyllanthus emblica* leaf extract-SNPs	Wistar Rat model	Hepatocellular carcinoma	Decreased hepatic lipid peroxidation, Na + /K + ATPase increased activity, decreased inflammation and	[70
	Dipalmitoyl-phosphatidyl choline-mediated Liposome-AgNPs	THP-1 cell line	-	H2AX histone phosphorylation and inhibition of Bcl-2 protein expression	[70]
	*P. aeruginosa*-based Au–Ag-alloy NP	A549 cell line	Metastatic lung	ROS-mediated cell killing	[71]
INP	HINP-Human albumin coated with iron nanoparticles-loaded with Dox	4T1 murine-xenograft model	Breast	Theranostic mechanism	[72]
	1 ml of magnetic fluid and17 quadrillion INPs	Human glioblastoma patients	Glioblastoma	Magnetic field-based a nanoactivator for thermic treatment	[73]
	IONP-bacteria derived magnetosome	U87-Luc and GL-261	Glioblastoma	Magnetic field-based elevated temperatures of 41–45 °C for cancer cell killing without side effects	[74]
	SPIO NPs-Dox-rhodamine B isothiocyanate-transferrin	U252 MG	Brain tumour	Cell imaging and improved uptake of the drug led to cell apoptosis and autophagy	[75]
	IONPs functionalized with CpG	N9 microglia cells	Glioblastoma	Stimulate the immune response against cancer cells	[76]
	Multifunctional RNA-loaded magnetic liposomes	B16F10-OVA murine	Melanoma	Activates the dendritic cells-based immune therapy	[77]
	Superparamagnetic INPs-loaded-Hsp70	C6 glioma	Glioblastoma	Stimulate tumour-specific CD8 + cytotoxic T cell	[78]
	Fe3O4-ZnO-CEA	C57BL/6 mice	-	Enhanced tumour antigen-specific T-cell responses, delayed tumour growth	[79]
	Fe3O4–Au-MB- PDPPA-1	EMT-6 cells- BALB/c mouse	Murine mammary carcinoma	Immunosuppressive TME and the activation of CTLs	[80]
	IO@FuDex^3^- fucoidan–dextran	4T1 cancer xenograft tumour model	Triple-negative breast	Magnetic field facilitating the active enrichment of immune cargos at the tumour site	[81]
ZnO-NP	ZnO-taxifolin-loaded NP	MCF-7 cell line	Breast	Apoptosis and the production of reactive oxygen species	[82]
	ZnO-Chitosan-cellulose hydrogel conjugated	A431 cells	Human skin carcinoma	ROS-mediated cell killing	[83]
	ZnO-PEG coated	MCF-7	Breast	Increased intracellular ROS when exposed to UV light	[84]
Silica-NPs	MSNPs loaded with curcumin and colchicine	HCT-116 cells	Colon carcinoma	Increased p53, caspase-3, and Bax expression, but inhibition of Bcl-2	[85]
	MSNPs—combined with biomass-derived GQDs	Mice	Subcutaneous and orthotopic HCC tumours	It produces NO as well as alleviates HCC hypoxia via NO	[86]
CaO_2_	CaO_2_-loaded polydopamine nanoparticles	4T1 breast cancer cells	Triple-negative breast	Downregulating the HIF-1α, which further downregulated the GLUT1 and LDHA	[87]

### Silver nanoparticles

Apart from GNP, silver NPs (SNPs) are commonly used for drug delivery against cancer and other diseases as SNPs release a high amount of ROS to kill the organisms. Biogenic synthesis of SNP was achieved by *Pinus roxburghii* extract where the synthesized SNPs showed apoptosis-mediated DNA damage and caspase-activated cell killing of lungs and prostate cancer cells [69]. *Phyllanthus emblica* leaf extract-based SNP was synthesized, and it showed potent anticancer activity against hepatocellular carcinoma (HCC) [88]. Since chemically synthesized SNPs are toxic, biogenic synthesis using plant extract/metabolites is always preferred. In another study, liposome-SNP nanocomposite increased the ROS generation leading to DNA damage in the cancer cells. Thereafter, Bax and Bcl2-mediated cytochrome c release was observed which further led to the caspase-activated killing of cancer cells [70]. Using metabolites, pyoverdine, and pyocyanin, from *P. aeruginosa* an alloy of gold and silver NP were synthesized with varying size from 20 to 2000 nm. These alloys showed a significant anticancer effect with an IC_50_ in the range of 10–35 µg/ml with faster internalization of smaller NPs (20–40 nm) inside the tumour cells [71]. The optimized morphology of AgNPs and porous silicon Bragg (PSB) mirror are combined to form a Surface-enhanced Raman scattering (SERS)-based signal enhancement detector known as Ag_2_O-Ag-PSB [89]. Here, Ag_2_O prevents natural oxidation of AgNPs and provides solid localized surface plasmon resonance where the intense SERS signal was measured at 300 °C.

### Iron nanoparticles

Iron NPs (INPs) are approved by FDA for cancer detection, hyperthermia-based cancer therapy, and delivery in case of anaemia deficiency [90]. INPs are employed to study cancer tumours by MRI [90]. EGFR antibodies, short peptides like RGD tripeptide, and aptamers are used to functionalize INPs. They have been offered as a treatment for cancers of the kidney, stomach, liver, breast, colon, and brain [89]. Human serum albumin (HSA)-coated and DOX-embedded INPs (D-HINPs ~ 50 nm) showed sustained release in both in vitro and in vivo studies through immunostaining and MRI, respectively [72]. HINPs accumulate in the nucleus and reduce tumour size > threefold compared to free DOX in the murine xenograft model of breast cancer. Interestingly, the pharmacokinetics studies showed that DOX uptake from D-HINPs is > 7.5-fold and > 3.5-fold higher than DOX and Doxil(a liposome-based DOX formula used in the clinic) after 4 h, respectively. NanoThermTM (patented by MagForce, USA), composed of 1 ml of magnetic fluid and 17 quadrillion INPs, is a commercial success for the focal treatment of solid tumours where the magnetic INPs are activated by a magnetic field using a nanoactivator [73]. Magnetotactic bacteria, known as magnetosomes, belong to a group of bacteria that can synthesize INPs covered by biological materials, thereby making the INPs completely biogenic with very low toxicity. These biogenic INPs were found to abolish the glioblastoma tumours when applied with an alternating magnetic field at an elevated temperature of 45 °C [74]. INPs are modified and functionalized with transferrin to achieve the maximum uptake of NPs on transferrin receptor-overexpressed brain tumour cell line U252MG for both imaging and therapy [75].

MNPs functionalized with CpG, when exposed to a magnetic field, were internalized and trafficked into the endosomes of N9 microglia cells, to deliver an immunostimulatory cargo for better antitumour efficacy [76]. Fe_3_O_4_ NPs, which can be loaded with multiple agents due to their large surface area, can deliver antigens into DCs with an inherent advantage of MRI-based application [77]. Superparamagnetic INPs loaded with heat shock protein 70 (Hsp70) stimulate tumour-specific CD8 + CTL responses by delivering immunopeptides from tumour lysates to DCs which results increased interferon (IFN)-γ levels in serum [78]. A core–shell MNP composed of Fe_3_O_4_-ZnO(~ 15.7 nm) was delivered with carcinoembryonic antigen (CEA) to DCs, ex vivo, where > 95% of DCs internalized a large number of MNPs within 1 h. Further, these MNPs allowed monitoring of MNP-labelled trafficking of DCs by MRI, in vivo, after hind footpads injection of C57BL/6 mice where in comparison with control groups, tumour growth was significantly inhibited in both C57BL/6 mice and the transgenic C57BL/6 mice which spontaneously expresses human CEA [79]. A TME-responsive and MNP-based nanocarrier was delivered with a short D-peptide antagonist of PD-L1 (named PDPPA-1), which could act as immune checkpoint inhibitor to modulate the immunosuppressive TME with subsequent activation of CTLs [80]. A fucoidan–dextran-based magnetic nano-agent (IO@FuDex3) showed strong magnetophoresis in response to increasing magnetic field, which facilitates the active enrichment of immune cells at the tumour site, thereby improving the anticancer efficacy and increasing the median survival of mice in a subcutaneous xenograft tumour model of 4T1 triple-negative breast cancer [81]. All of these demonstrate how INPs have the potential to become the standard of care for cancer treatment and cancer detection in the future because they have magnetic properties that can be controlled externally.

### ZnO nanoparticles

Zinc oxide (ZnO), one of the safest NPs, allows functionalization/conjugation of various molecules for drug delivery against cancers due to its low cost and eases of synthesis with high surface area [91]. Biogenic synthesis of ZnO NPs is cheaper and safer than most other metallic NPs [92]. ZnO-taxifolin-loaded NP, coated with a biodegradable polymer, showed sustained release and apoptotic cell death against the MCF-7 cell line with less toxicity compared to naked taxifolin, a natural polyphenol [82]. A hybrid hydrogel composed of histidine-conjugated chitosan, phyto-synthesized (naringenin, quercetin, and curcumin) ZnO NPs, and dialdehyde cellulose was prepared where drug loading capacities for all 3 polyphenols were ~ 90%. The ZnO NP formulation releases the drug at acidic pH of 5 which specifically delivered the drugs to TME, thereby increasing the anticancer efficacy > 30-fold in comparison with free polyphenols against human skin carcinoma A431 cells [83]. PEG-coated ZnO nanorods (PEG-ZnO-NRs), when loaded with pro-oxidant piperlongumine (PL) and/or coated with GNPs, showed increased efficacy of ROS production, thereby killing the breast cancer cell line MCF-7 by apoptosis under UV irradiation in PDT [84]. ZnO NPs used for triple-negative breast cancer (TNBC), increase ROS production which imbalances the redox-homeostasis to kill the MDA-MB-468 cancer cells without showing any cytotoxicity for normal cells [93]. Here 4 distinct Zn coordination compounds, synthesized from Zn-carboxylates & hexamethylenetetramine, are converted to ZnO NPs by ultra-sonication with oleic acid or heating of pure precursors in an air atmosphere to create NPs of size in the range of 20–350 nm.

### Silica nanoparticles

Silica NPs are mesoporous within a diameter range of 2–50 nm, and these mesoporous silica NPs (MSNP) are widely used for drug delivery, catalysis, adsorption, separation, etc. Two natural agents, curcumin and colchicine, were loaded on MSNPs, and efficacy was tested against several cancer cell lines, in vitro, (breast cancer cell MCF-7, colon carcinoma HCT-116 cells, lung cancer A-549 cells) where maximum efficacy was obtained against HCT-116 cells with an IC_50_ < 5 µg/ml showing apoptotic death with increased p53, caspase-3, and Bax expression, but inhibited Bcl-2 expression [85]. Biomass-derived GQDs combined with hollow MSNPs (hMSNPs) are used for fluorescent imaging and dual treatment of cancer via drug delivery and PDT. Although the addition of hMSNPs creates more toxicity, the GQDs-hMSNs solved dual purpose—drug delivery using fluorescein isothiocyanate (FITC) as a mock drug, and PDT treatment by using the GQDs as a photosensitizer which can induce singlet oxygen-mediated high ROS. Combined gas-radiotherapy (GT-RT) from a gold-capped MSNP was possible due to the loading of NOSH-aspirin which is a dual-donating cytotoxic gas producer—nitric oxide and hydrogen sulphide. X-ray irradiation along with drug delivery creates a unique GT-RT reduced subcutaneous and orthotopic hepatocellular carcinoma tumours with high biocompatibility [86].

### CaO_2_ nanoparticles

Hypoxia and high accumulation of lactic acid in the TME favour tumour development, maintenance, and metastasis. In an interesting study, calcium peroxide (CaO_2_)-loaded polydopamine nanoparticles modified with sodium hyaluronate (denoted as CaO_2_@mPDA-SH) can gradually accumulate in a tumour site. CaO_2_ exposed in an acidic microenvironment can consume the lactic acid with oxygen generation simultaneously with reduced tumour size in animal models [87]. These data are correlated with downregulating the hypoxia-inducible factor-1α (HIF-1α), which further downregulated the glycolysis-associated enzymes including glycolysis-related glucose transporter 1 (GLUT1) and lactate-dehydrogenase A (LDHA); thereby, this peroxide NP can modulate the TME.

## Dendrimers

**Fig. 3:**
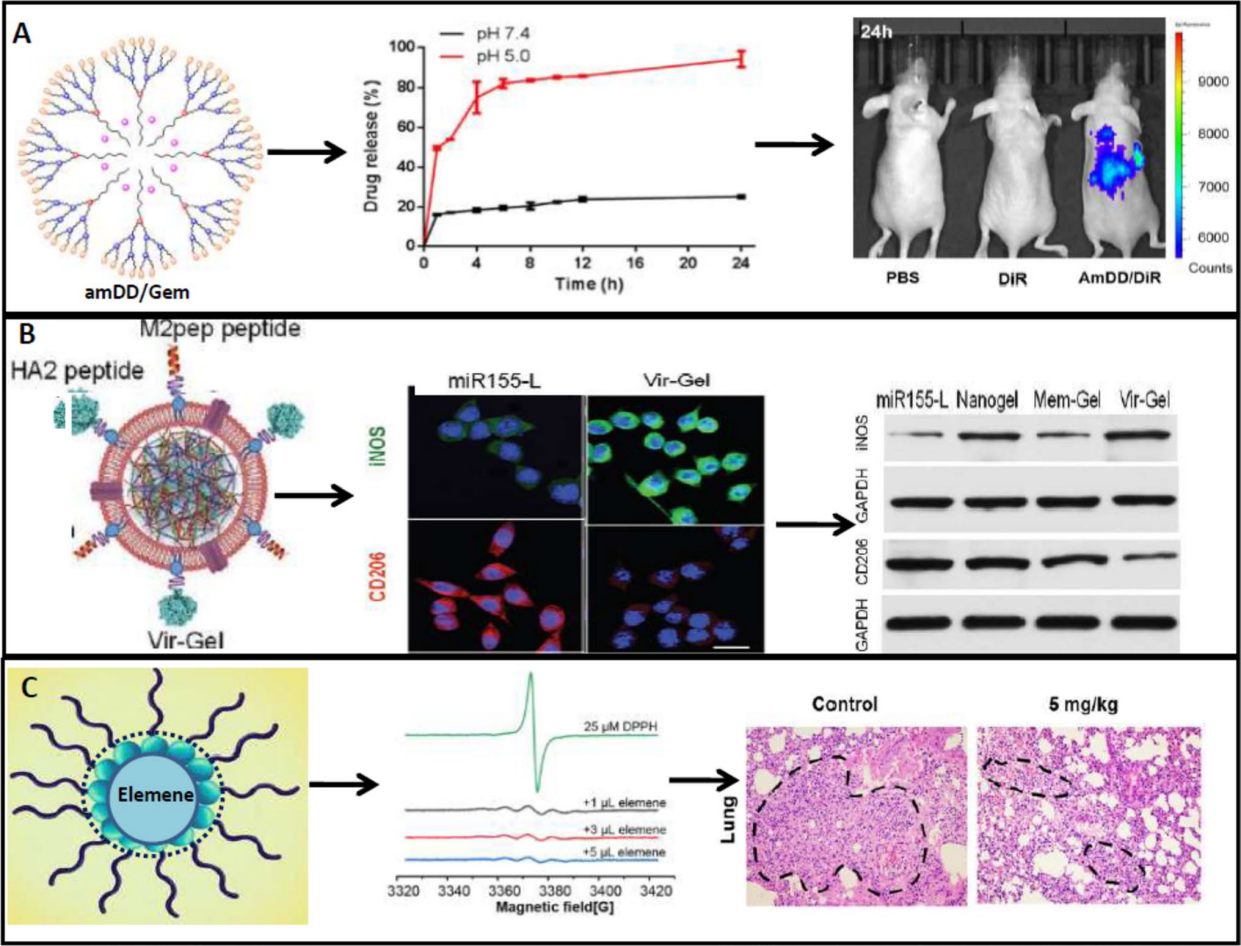
Dendrimer, nanogel, and nanoemulsion-based drug delivery for cancer. **A** Amphiphilic dendrimer (amDD)-based gemcitabine delivery shows increased drug release at pH 5.0 and more accumulation of drug in tumour model of mice measured by fluorescence. **B** Virus-mimicking erythrocyte membrane-coated miRNA-155 loaded nanogel (Vir-Gel)shiftsM2 phenotype to an anti-tumour M1 phenotype in glioblastoma by increased expression of iNOS and decreased expression of CD206. **C** Nanoemulsion of elemene shows strong antioxidant effect measured in ESR spectrum and decreased metastasized nodules in the lung with 5 mg/kg dose in mice. The **A** and **C** were adapted from references [98] and [101], for which copyright permission is not required. The **B** was reproduced/adapted from reference [102] with permission from [John Wiley and Sons], all right reserved

Dendrimers are highly branched tree-like polymeric architects with well-defined monodispersed nanostructure, a high degree of surface functionality, and low immunogenicity. The three basic elements of dendritic polymers are a centralized core, a middle or interior portion made up of repeated branches, and an outside shell defined by terminal functional groups. There have been numerous applications on dendrimer-mediated targeted delivery with hydrophobic and hydrophilic dendrimers as well as phospholipids to create a dendrimer/lipid nanocomplex [94]. The branching of dendrimers is particularly valuable because they can give variable and large quantities of surface fictionalization groups for chemical modifications. The in vitro targeting capacity of partially acetylated 5-polyamidoamine dendrimer (PAMAM-AcG5) in HeLa cells was tested when it was conjugated with biotin as the targeting moiety [2]. The AcG5-biotin-FITC multifunctional conjugate demonstrated much better cellular absorption than the conjugate without biotin. The PAMAM's hyper-branched properties, possibly, allow the dendrimer to breach the cell membrane of cancer cells and transport a large amount of cargo. The dendrimer's low pKa value reduces the non-specific binding of blood proteins and allows sustained drug release. The dendrimers with changeable surface functionality, multi-valency, water solubility, monodispersity, and large internal space for drug, and small molecule encapsulation make them suitable for various applications [95]. Due to its advantages standard anticancer drugs like DOX are also delivered through dendrimers where controlled release, better penetration, improved bioavailability, and reduced cardiac toxicity were associated with DOX-dendrimer delivery [96]. Gemcitabine, a nucleoside analogue and the first line of drug for pancreatic cancer, is severely limited in its efficacy due to its instability and poor cellular uptake. An aliphatic gemcitabine (gem) prodrug and a small amphiphilic dendrimer were encapsulated together (AmDD/Gem) with ~ 33% drug loading capacity [97]. Compared to standard gemcitabine, the AmDD/Gem (~ 20 nm) showed a nearly 2.5-fold decrease in tumour volume in mice models of pancreatic cancer with reduced side effects indicating the efficacy of dendrimer-based delivery of gemcitabine. The PAMAM dendrimers modified with PEG chains and conjugated with Flt-1 antibody (a receptor for VEGF) reduce the tumour size by 30–50% in human xenograft CFPAC-1 pancreatic tumours model where normal gemcitabine is ineffective [98]. The delivery of siRNA/dendrimer with the negatively charged segment of the targeting peptide via electrostatic interactions led to small and stable nanoparticles which were able to protect siRNA from degradation and successful delivery in vitro and in vivo [99]. Downregulation of cancer in prostate cancer cells PC-3 and Hsp27 and increased uptake of siRNA in PC-3 xenograft nude mouse model confirmed the efficacy of dendrimer-based delivery. Several other dendrimers, such as poly-L-glutamic acid polyesters (PGLSA-OH), poly(2,2-bis(hydroxymethyl)propionic) acid (bis-MPA) scaffold dendrimers, poly(propyleneimine) (PPI), poly-L-lysine (PLL) scaffold dendrimers, glycodendrimers, carbosilane (CBS) dendrimers, and phosphorus dendrimers, are emerging as drug delivery vehicles in cancer [100]. In Fig. 3, the dendrimer-, nanogel- and nanoemulsion-based anticancer drug delivery with efficacy and target was shown from the published data with permissions.

## Nanogels

Nanogels are sub-micron polymer-based crosslinked hydrogel particles [103] where at the convergence of NPs and hydrogel association, these intricate networks of polymers provide a unique possibility of drug encapsulation and delivery. The difference between nanogels and liposomes is that nanogels are from polymers, whereas liposomes are mainly from lipid or lipid vesicles. Smaller particle size (10–200 nm), biodegradability and/or biocompatibility, prolonged blood circulation time, increased amount of drug or enzyme loading, and protection of encapsulated molecules from the body's immune system are all characteristics of an ideal nanogel drug delivery carrier [104]. Drug loading in nanogels can be done efficiently afterwards when the nanogels are swelled and equilibrated in water or biological fluid; hence, nanogels can be manufactured or synthesized even without the drug being loaded [105, 106]. Currently, stimuli-responsive nanogels are in limelight, which responses against various stimuli like pH, ROS, redox, hypoxia, etc., and releases the drug at the target sites [107]. Changhuan Zhang et al., 2021 developed a hypoxia- and enzyme-responsive PEGylated hyaluronic acid nanogel which releases the cytokine, interleukin- 12 (IL-12), as a drug in the hypoxia condition of melanoma cancer cells [108]. Importantly, in presence of 500 U/ml of hyaluronidase, the drug release was significantly higher indicating that the nanogel is also enzyme-responsive. In another study, virus-like membrane-coated nucleic acid nanogel Vir-Gel is entrapped with miRNA-155. This nanogel formulation allows the miRNA to stay in circulation longer which further enhanced the anti-tumour efficacy. The nanogel can reprogramme the microglia and macrophage from the pro-oncogenic M2 phenotype to anti-oncogenic M1 phenotype [109]. One hybrid gel formulation combined with hydrogel and nanogel was prepared for thermo- and ROS-responsive release of drug. Here nanogel is embedded with LY3200882, a TGF-β inhibitor, while hydrogel was loaded with regorafenib, an antitumour drug. Cancer cells having elevated temperature as compared to normal cells trigger the thermo-responsive effect of hydrogel. Regorafenib increases ROS production which further stimulates the release of LY3200882 which inhibits the TGF-β, thereby increasing the anti-cancer potency [102]. Cisplatin embedded with alginic acid nanogel (AL/CDDP) for ovarian cancer [110], while paclitaxel-loaded mucoadhesive nanogel was used for cervical cancer treatment [111]. The AL/CDDP-nanogel showed high in vivo efficacy when delivered intraperitoneally against an ovarian cancer mouse model using ovarian cancer cell lines with KRAS mutations (ID8-KRAS: KRAS^G12V^). For paclitaxel-loaded hydrogel poly(acrylic acid) is used as the polymeric agent and in vivo efficacy was supported by the increased retention time of the nanogel in the vagina and reduced tumour growth. A pH-responsive fluorescence-enhanced nanogel was prepared for breast cancer therapy where auraptene (AUR), a natural bioactive prenyloxy coumarin, and cisplatin are added as drugs [112]. In vivo experiments showed that the nanogel has reduced systemic toxicity and better antitumour activity. These all circumstances indicate that nanogel-based drug delivery is one of the important strategies for cancer treatment. Drug delivery against different cancers with nanogel, nanoemulsion, and nanoshell carrying various anticancer drugs with the possible mechanism of actions and targets is summarized in Table 3.

**Table 3:** Drug delivery against different cancers with nanogel, nanoemulsion, and nanoshell carrying various anticancer drugs along with the possible mechanism of actions and targets

Type of nanoparticle	Composition or drug	Study model	Type of cancer	Possible molecular mechanisms	References
PEGylated Hyaluronic Acid Nanogel	Cytokine: Interleukin-12 (IL-12)	Melanoma cancer cells	Melanoma	Hypoxia- and enzyme-responsive, releases IL-12 in hypoxic conditions, enzyme-responsive release with hyaluronidase	[108]
Hyaluronic acid Virus-like Membrane-Coated Nucleic Acid Nanogel (Vir-Gel)	miRNA-155			Enhances miRNA circulation, reprograms microglia, and macrophages	[109]
Hydrogel + Nanogel	TGF-β Inhibitor: LY3200882 (embedded in nanogel), Antitumour Drug: Regorafenib (loaded in hydrogel)			Thermo- and ROS-responsive release, elevated temperature triggers hydrogel's release, increased ROS production stimulates nanogel's release, increases anti-cancer potency	[102]
Alginic Acid Nanogel (AL/CDDP)	Cisplatin	Ovarian cancer mouse model with KRAS mutations (ID8-KRAS: KRAS G12V)	Ovarian Cancer	High in vivo efficacy when delivered intraperitoneally, effective against ovarian cancer with KRAS mutations	[110]
Mucoadhesive Nanogel	Paclitaxel		Cervical Cancer	Used for cervical cancer treatment, increased retention time in the vagina, reduced tumour growth	[111]
pH-Responsive Fluorescence-Enhanced Nanogel	Auraptene (AUR) and Cisplatin		Breast Cancer	Reduced systemic toxicity, better antitumour activity, pH-responsive, enhanced fluorescence	[112]
Elemene Terpene-Loaded Nanoemulsion	Elemene terpene	Mice model	Triple-Negative Breast Cancer (TNBC)	Acts as an antioxidant, reduces ROS levels, stabilizes HIF-1α, decreases tumour size	[113]
Neobavaisoflavone Nanoemulsion	Neobavaisoflavone (natural flavonoid)			Potent antioxidant and anti-inflammatory effects, inhibits TGF-β/SMAD pathway, reduces extracellular matrix deposition	[101]
γ-Tocotrienol (γ-T3)-Based Paclitaxel Nanoemulsion	Paclitaxel	Pancreatic cancer cell line	Pancreatic Cancer	Improved efficacy, reduced IC50, PEGylation enhances effectiveness	[114]
Gingerol-Loaded Nanoemulsion	Gingerol	TNBC cell line	Triple-Negative Breast Cancer (TNBC)	Increases solubility and stability of gingerol, further evaluation needed for in vivo efficacy	[115]
Chitosan-Modified Cationic Nanoemulsion	Gambogic acid (GA) + siRNA (targeting XIAP)	A459 lung cancer cell line	Lung Cancer	Increased residence time in lungs, increased apoptosis	[116]
Self-Nanoemulsifying Formulation	Genkwanin (O-methylated flavonoid)	Mice model of colorectal cancer	Colorectal Cancer	Enhanced bioavailability, significant reduction in colon tumours	[117]
Nanoshell with Magnetic Core and Biodegradable Shell	5-Fluorouracil			Regulated release of 5-fluorouracil	[118]
Gold Nanoshells	Gold	Prostate cancer cells	Prostate Cancer	Conversion of infrared light into heat, thermal ablation of malignant cells	[119]
Gold Nanoshells	Rap2b siRNA	HCT116 colorectal cancer cells	Colorectal Cancer	Increased anticancer efficacy of adriamycin (ADR), laser-induced release of siRNA	[120]
Hollow Mesoporous MnO_2_ (H-MnO_2_) Nanoshells	Docetaxel and Cisplatin (TP)	Metastatic Cancer	Metastatic Cancer	Decomposition in acidic tumour microenvironment, release of TP drugs, reduced tumour hypoxia, and HIF-1α expression	[121]
Polydopamine Nanoshells	Oxaliplatin (OXA) and Gedatolisib	Hepatocellular Carcinoma	Hepatocellular Carcinoma	Reversal of drug resistance, co-delivery of OXA, and Gedatolisib	[122]
Gold Nanoshells	EML4-ALK fusion gene and microRNA-301	Non-Small Cell Lung Cancer (NSCLC)	Non-Small Cell Lung Cancer (NSCLC)	Combined effect of gene therapy, increased stability of genes inside nanoshells	[123]

## Nanoemulsions

Nanoemulsions are thermodynamically stable isotropic systems in which two immiscible liquids (generally water and oil) are combined to produce a single phase using variable amounts of surfactants or its mixture to create droplet diameters ranging from 0.1 to 100 µm [124]. Therefore, a heterogeneous system in which the oil phase is disseminated as droplets in an aqueous phase and stabilized by an emulsifying agent is referred to as a nanoemulsion. The nanoemulsions have been synthesized with specific parameters (e.g. size, surface charge, prolonged blood circulation, target-specific binding ability, and imaging capability) where the angiogenesis inhibitors can be encapsulated to reduce toxicity and improve therapeutic efficacy against tumours [125]. Elemene terpene-loaded nanoemulsion prepared and its effect on the metastasis of triple-negative breast cancer (TNBC) was evaluated. This formulation acts as an antioxidant which significantly reduced the level of ROS that destabilizes the hypoxia-inducible factor-1α (HIF-1α) and consequently, tumour size was decreased in the mice model [113]. When neobavaisoflavone, a natural flavonoid, was trapped in lecithin-based nanoemulsion it showed a more potent antioxidant and anti-inflammatory effect than the naked isoflavonoid. It decreased the deposition of extracellular matrix near cancer cells by inhibiting the TGF-β/SMAD pathway [101]. Further, γ-tocotrienol (γ-T3)-based paclitaxel nanoemulsion was formulated with the help of PEGylation. The average size and surface charges were ~ 220 nm and − 42 mV, respectively. It showed improved efficacy in a pancreatic cancer cell line, where IC50 was found to be ~ 0.5 μM which is twofold lower for tocopherol-based paclitaxel (PTX) nanoemulsion than the drug alone [114]. In addition, gingerol-loaded nanoemulsion was tested against the TNBC cell line in both murine and humans. By nanoemulsion, gingerol solubility in water and stability was increased. However, this study was performed in vitro only further evaluation in the animal model is necessary to confirm the efficacy and stability [115]. The chitosan-modified cationic nanoemulsions were prepared which are composed of gambogic acid (GA), a naturally occurring anticancer agent, and siRNA which are targeted against X-linked Inhibitor of Apoptosis Protein (XIAP) which are potent inhibitors of caspases/apoptosis [116]. Generally, most drugs/siRNAs have a long residence time in the lungs because of pulmonary delivery thereby having greater therapeutic effects compared to systemic administration. This was confirmed by increased apoptosis against A459 lung cancer lines in vitro. A self-nanoemulsifying formulation composed of maisine CC, labrasol ALF, and transcutol HP in a weight ratio of 20:60:20 was entrapped with genkwanin, a naturally occurring O-methylated flavonoid, for delivery against colorectal cancer [117]. The nanoemulsion showed > 3.5 fold more bioavailability than standard genkwanin. In parallel, colon tumours were reduced significantly for the nanoemulsion in the mice model of colorectal cancer.

## Nanoshells

Nanoshells are made out of a sphere-shaped dielectric core made of silica or other similar materials that are surrounded by a metal coating such as gold or silver [126]. These nanoshells transform plasma-mediated electrical energy into light energy, and they can be optically adjusted using UV-infrared emission/absorption arrays. Therefore, nanoshells are helpful in photothermal cancer therapy mediators because they are efficient at converting absorbed radiation into heat and are stable at a wide range of temperatures. Even though their applications are constrained by their huge diameters, nanoshells are desired since their application is free of heavy metal toxicity. For the regulated release of 5-fluorouracil, nanoshell particles with a magnetic core of carbonyl iron and a biodegradable polybutylcyanoacrylate (PBCA) shell have been generated [118]. Antibodies or other biomolecules are attached to the gold nanoshell surface in cancer applications to direct it to the tumour site, and it has been used to destroy/kill prostate cancer cells. Preclinical experiments by Lal and colleagues showed that gold nanoshells may transform infrared light into heat at tumour locations, killing malignant cells quickly [119]. The gold layer thickness can be adjusted such that the nanoshell can be selectively triggered by irradiating the tissue with near-infrared light to perform targeted therapeutic thermal ablation. In mice, the method was recently used to remove transmissible venereal tumours, tumours that arise more commonly in dogs from the dysregulated growth of histocyte cells. Rap2b, a P53 target, sensitizes HCT116 colorectal cancer cells to apoptosis induced by adriamycin (ADR), indicating that Rap2b promotes ADR resistance in cancer cells. Delivery of Rap2b siRNA-loaded gold nanoshells in the presence of ADR increases the anticancer efficacy of ADR in vitro and in vivo [120]. Here, the laser-induced release of siRNA from nanoshell was found to be the reason for increased efficacy. In another study, hollow mesoporous MnO_2_ (H-MnO_2_) nanoshells were loaded with chemotherapy agents docetaxel and cisplatin (TP) and then their efficacy was studied against metastatic cancer [121]. The obtained H-MnO_2_-PEG/TP nanoshells decomposed in the acidic tumour microenvironment, releasing the loaded drugs (TP) causing reduced tumour hypoxia and hypoxia-inducible factor-1α (HIF-1α) expression. Also, the uptake rate of MnO_2_nanoshellsis, in vivo, is much higher than standard TP. Drug resistance against hepatocellular carcinoma can be reversed by polydopamine nano-shells coloaded oxaliplatin (OXA) and gedatolisib-mediated (a PI3K/mTOR inhibitor) drug delivery [122]. The hollow nanoshells in MnO_2_ and polydopamine also allowed more drug encapsulation. Gold nanoshell-based delivery for echinoderm microtubule-associated protein-like 4-anaplastic lymphoma kinase (EML4-ALK) fusion gene and microRNA-301 against NSCLC showed combined effect than monotherapy where a mutation in these genes is associated with this cancer [123]. This kind of delivery, equivalent to gene therapy, was much more effective due to the increased stability of genes inside gold nanoshells.

## Micelles

Micelles are colloidal particles that range in size from 5 to 100 nm. Micelles are made up of amphiphiles or surfactants that have two different regions: a hydrophilic head and a hydrophobic tail. Micelles are different from emulsions because they are thermodynamically more stable and there is a more dynamic exchange of surfactants in and out of the micelles. The amphiphiles exist as monomers in true solution at low concentrations in an aqueous media, but when concentration rises, aggregation and self-assembly occur within a restricted concentration window, and micelles develop. Due to their particular structural composition, which is defined by a hydrophobic core sterically stabilized by a hydrophilic shell, block copolymer micelles can improve the solubility of hydrophobic molecules. Depending on their physicochemical qualities, the hydrophobic core acts as a reservoir into which drug molecules can be integrated via chemical, physical, or electrostatic interactions [127]. Micelles generated by polymeric molecules are thought to be structurally more stable than micelles formed by low molecular weight substances [128]. Polymeric nanoparticles (PNPs) are solid micelles with particle sizes ranging from 10 to 1000 nm. PNPs, also known as nanospheres, nanocapsules, and polymer micelles, were the first polymers to be used in drug delivery systems. A micelle synthesized with phenylboronic acid was loaded with DOX and irinotecan as an anticancer drug. The drug-loaded micelles (~ 30–40 nm) reduced the tumour size to normal within 16 days of treatment with no systemic toxicity [129]. Sunitinib, a first-line therapy for renal cell carcinoma treatment, is limited in use due to its toxicity, side effects, and low bioavailability. Sunitinib was loaded on sialic acid-poly (ethylene glycol)-ibuprofen (SA-PEG-IBU) amphipathic conjugate to create a nano-micelle [130]. Enhanced cancer cell uptake was observed due to the presence of an E-selectin receptor on the membrane of cancer cells. This allows increased binding with sialic acid present in nanomicelle and also had a superior therapeutic effect and lower toxicity than sunitinib alone. For a combination of immunotherapy and chemotherapy, pH/reduction dual-responsive co-delivery micelles were developed using chitosan-coated hyaluronic acid micelles that deliver DOX and programmed death-ligand 1 small interfering RNA (siPD-L1) together [131]. In vivo studies showed that the micelle exhibits a significantly stronger anti-breast cancer effect due to increased uptake of DOX mediated by CD44-hyaluronic acid interaction followed by increased generation of CD4 + /CD8 + T cells by silencing PD-L1 expression.

## Hydrogels

**Fig. 4:**
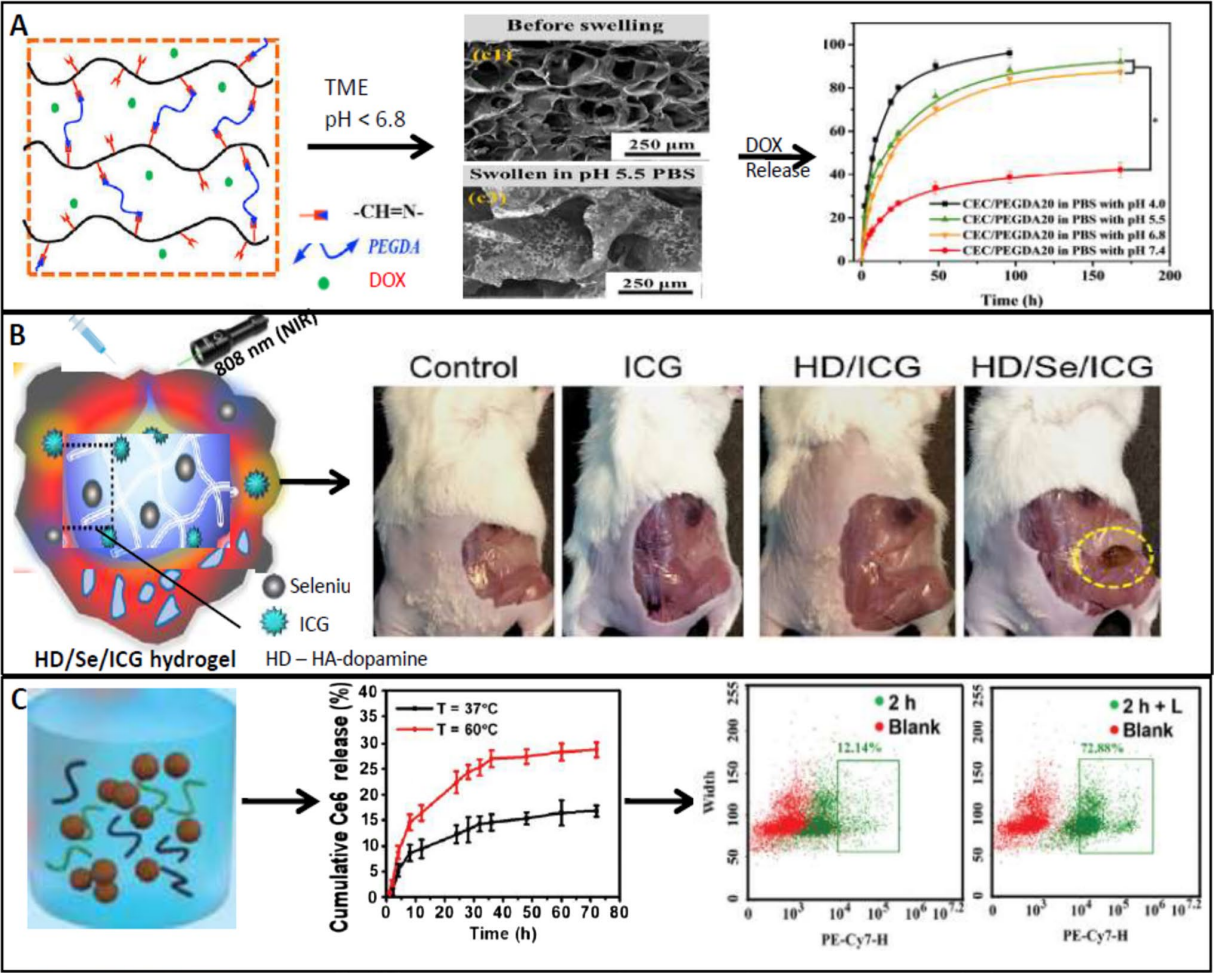
Application of stimuli-responsive hydrogels in anticancer drug delivery. **A** A pH-responsive polysaccharide-based hydrogel with swollen properties at low pH in TME with increased DOX release for hepatocellular carcinoma. **B** A photo-responsive cross-linked hydrogel composed of HA, dopamine, and Se metal coordination (HD/Se/ICG) carrying indocyanine green (ICG) provided tumour growth inhibition due to intra-tumoural injection and NIR irradiation at 808 nm. **C** An agarose-based photothermal-responsive hybrid hydrogel composed of photosensitizer Ce6 releases the drug at elevated temperature measured by FACS. The **A** and **B** were reproduced/adapted from references [133] and [136] with permission from [Elsevier], copy rights reserved. The **C** was reproduced/adapted from references [135] with permissions from [Royal Society of Chemistry], copy rights reserved

In 1960, the term hydrogel was first coined by Wichterle and Lim when a synthetic biocompatible material poly-2-hydroxyethylmethacrylate (PHEMA) was prepared in a gel form that became useful for contact lens applications. Hydrogels are generally described as a cross-linked, water-swollen polymeric network created by the chemical conjugation of one or more monomers which can absorb large amounts of water that may exceed thousands of times of polymers dry-weight. These hydrogels become practically more useful due to their affinity for biological fluids. The biggest advantage of hydrogel-based drug delivery is their stimuli-responsive (pH, temperature, light, ionic strength, magnetic force, etc.) conformational change at the site of action. Due to this feature hydrogels are routinely combined with implants for antibacterial applications in wounds/infections. A gelatine-based hydrogel was crosslinked with cationic quaternary ammonium salt to provide high biodegradability, good mechanical properties, and bactericidal activity in the infective model of femoral fracture of rats [123]. Chitosan is one of the most used polymers to produce pH-responsive hydrogel along with its derivatives like N-carboxyethyl chitosan (CEC). A DOX-loaded hydrogel with self-healing capacity (20 mm diameter and 1 mm thickness) was created, by covalent Schiff-base linkage using amine groups from CEC and benzaldehyde groups from PEG diacrylate (PEGDA), for application against hepatocellular carcinoma [132]. It was observed that the hydrogel degraded in PBS of pH 5.5 much faster than that in PBS of pH 7.4, after 48 h, thereby allowing selective DOX release in TME. The pH-responsive hydrogels have become an important tool for the oral delivery of anticancer drugs to counter the pH variations of the gastrointestinal tract. A hydrogel was planned for intestinal delivery of amifostine (FDA-approved radioprotective agent) through polycaprolactone-grafted poly(methacrylic acid-co-ethyl acrylate) (PCL-g-MAC)-based hydrogel. The hydrogel provided better stability of amifostine from gastric degradation since at pH 7.4 > 70% drug release was observed with 0.5 h, but at pH 1.2 the release is only 35% even after 5.5 h [133]. Further, in vivo studies showed this increased stability is due to inhibition of hematopoietic cell apoptosis during γ-radiation. Photosensitive hydrogels as a consequence of light exposure (UV, visible, or NIR) undergo modifications such as free-radical-based polymerization reaction, chemical cleavage, and/or isomerization, and changed volume due to swelling or shrinkage, etc. Using indocyanine green (ICG) (a photo-thermal agent) a sodium selenite (Se)-directed, crosslinked and hyaluronic acid-dopamine, loaded hydrogels were used for breast cancer applications [134]. Due to NIR irradiation, ICG allows both in vitro and in vivo photo-thermal activity (PDT) against MDA-MB-231 breast cancer cell lines, and BALB/c nude mice with tumour where in a 7^0^C increased temperature range the drug release was observed optimally. A biocompatible agarose-based hydrogel was prepared by incorporating chlorin e6 (Ce6) as a photosensitizer, sodium humate (SH) as a photothermal agent, and manganese oxide (MnO_2_) as the catalytic decomposer of H_2_O_2_ and modulation of hypoxia at the tumour microenvironment which are associated to treatment resistance [13]. Under NIR irradiation, SH by PDT degrades the agarose gel forming H_2_O_2_ which is further decomposed into O_2_ and then converted into ^1^O_2_ (by the released Ce6) which is responsible for a strong anticancer activity against 4T1 BALB/c mice, in vivo. The hydrogels that are magneto-sensitive are generally composed of hydrogels incorporated with iron oxide NPs which under exposure to increasing doses of magnetic field significantly increase local temperature promoting a therapeutic efficacy by triggering increased drug release from thermo-sensitive hydrogels. A magnetic-sensitive hydrogel which was composed of PEGylated Fe_3_O_4_ NPs and α-cyclodextrin is stabilized by a PEGylated phospholipid which is encapsulated with doxorubicin and paclitaxel [135]. In vivo, experiments against 4T1-tumour bearing *Balb/c* mice showed improved survival rate and reduced tumour recurrence of animals due to facilitated local delivery of sol-to-gel transition of the hydrogel under the applied magnetic field. Application of different stimuli-responsive hydrogels (pH-, photo- and magneto-responsive) in anticancer drug delivery is shown in Fig. 4 with data from published papers with permissions.

## Microneedle

The concept of microneedle (MN) technologies was introduced by Pistor in the 1970s as a transdermal drug delivery device, but the term MN was first coined in 1998[137]. MNs are comprised of a patch as the base support attached with micron-sized needles where several hundred-micrometre squares of microprojections form the MNs resembling honeybee comb structures with length, width, and thickness in the range of 500–900 μm, 50–250 μm, and 1–25 μm, respectively. One of the important features of MN-based delivery is that it works as a painless self-attachment where micron-size needles are loaded with vaccines, antibodies, drug molecules, proteins, genes, etc. Further, MNs are mechanically stronger drug delivery systems that can avoid first-pass metabolism. A vaccine against breast cancer was prepared by spray drying technique which contained whole cell lysate breast cancer cells as a source of tumour-associated antigens. The MN-sized particle (1.5 μm) resembled the pathogenic cancer cells, thereby helping in better antigen presentation and leading to improved activation of the immune response. This particulate MN-based vaccine which was delivered via skin using metal MNs was visualized by methylene blue staining and confocal microscopy. MNs created aqueous channels (50 ± 10 μm long) to deliver the vaccine to the skin layers where a higher concentration of B and T cell (CD4 + and CD8 +) populations & serum IgG, IgG2a, were measured in the vaccinated animals compared to the control animals [138]. More importantly, MN-based particulate vaccine delivery showed five times more tumour suppression than the non-vaccinated animals. In another study vaccine particle was prepared by spray drying method from tumour cell lysates of mouse ovarian cancer cell lines and delivered transdermally alone or in combination with IL-2 and IL-12. The delivery was done using AdminPen™, a metallic MN device, and efficacy was compared with an oral delivery route which delivered the vaccine by Aleuria aurantia lectin, a microfold (M)-cell targeting ligand, for targeted uptake of these M-cells present in Peyer's patches of the small intestine [139]. When the vaccine was delivered by combination routes along with IL-2 & IL-12 there was ~ 9 times more tumour suppression than the control vaccine NP alone. Further, increased serum IgG1, IgG2a, and IgG titres and activation of CD4 + T-cell, CD8 + T-cell, and NK cells provide direct evidence of both humoral and cellular immune response in MN-delivered vaccine. MN patches were prepared to deliver photosensitizer IR820 and chemotherapy agent cisplatin for combination chemo-photodynamic therapy against 4T1 bearing breast cancer model of Balb/c mice where tumour volume was reduced ~ 3.5 fold after 15 days [140]. Due to a microemulsion and microneedle-based delivery system, there is a ~ fourfold increased cutaneous and percutaneous delivery of celecoxib leading to a ~ 3.3-fold reduction of IC50 of celecoxib against MCF-7 cells [141]. All these data provide evidence that transdermal delivery of vaccines/drugs through MNs is more advantageous due to better on-site delivery leading to immune cell activation.

## Nanofiber

Nanofibres are synthesized by electrospinning techniques which use high voltage for conversion of liquid polymers into solid fibres of diameter ranging from nanometres to micrometres. By this method we can synthesize biodegradable, natural, synthetic, or non-degradable fibrous tubes or mats after slow and continuous drying of the polymers by solvent evaporation during electrospinning. The viscosity and concentration of polymer solution determine the spinnability of the preferred biomolecules with the possibility of synthesizing fibres or not, whereas other factors (applied voltage, flow rate, conductivity, solvent volatility, collector shape, and capillary distance, etc.) determine the shape and geometry of nanofibres. Drug loading and time-dependent drug can be very efficiently controlled by nanofibres. When microsol-electrospinning was used to load VEGF in electrospun nanofibres < 40% of VEGF was released in the initial 48 h, followed by sustained release over 4 weeks giving a long-term efficacy. The VEGF encapsulated in the hyaluron-based polymer was found to be released in a durable way for angiogenesis in the fibrous layer and bone defect area due to nanofiber-based delivery [142]. A nanofiber was designed by self-assembling amphiphilic peptides where the peptides were crosslinked with DOX (length ~ 175 nm, diameter ~ 12 nm). The nanofiber assembly was delivered in triple-negative breast cancer cells(TNBC) where matrix metalloproteinase-9 (whose levels are elevated) cleaves the peptide-drug conjugate to release the drug doxorubicin. Further, the use of super magnetic iron oxide NPs (SPIONs) in the formulations enabled the samples to be sensitive to the magnetic field. The formulation showed ~ tenfold decreased IC_50_for DOX against TNBC than normal DOX delivery [143]. A polylactic acid (PLA)/nanocellulose (NC)-based nanofiber was created with magnetic (M) NPs to incorporate 5-FU and curcumin (CUR). Two nanofibres were obtained PLA/M-NC/5-FU/CUR and M-PLA-co-NC/5-FU/CUR where in the 2nd nanofiber self-assembly of the polymer was used [144]. Interestingly the nanofiber showed both anticancer (HePG-2, MCF-7, and HCT-116 cell lines) and antibacterial (Streptococcus, Bacillus subtilis, Klebsiella pneumonia, and Escherichia coli bacteria) effects where nanofiber micelles M-PLA-co-NC/5-FU/CUR revealed slower release, higher antibacterial, and antitumour efficacy than the PLA/M-NC/5-FU/CUR. All these results show that sustained along with stimuli-based (like magnetic field) drug release is an advantage of nanofiber-mediated anticancer delivery.

## Exosomes

Exosomes, a kind of extracellular vesicle (IVs), are enclosed within a single outer membrane and are secreted by all cell types. They have been found in plasma, urine, semen, saliva, cerebral spinal fluid, breast milk, serum, tears, lymph, bile, etc., and are within a size range of 30–150 nm [145]. Exosomes participate in cell–cell communication, and cell maintenance, and more importantly, it can stimulate immune responses by acting as antigen-presenting vesicles. Exosomes are currently an ideal drug delivery platform due to their low immunogenicity, easy modification/engineering, high biocompatibility, and natural carrying capacity of drugs like in a layered lipid vesicle. Targeted delivery of antigens & adjuvants to DCs in vivo represents an important approach for the development of cancer vaccines. DC vaccine composed of human neutrophil elastase and hiltonol (a TLR3 agonist) was loaded into α-lactalbumin-engineered breast cancer MDA-MB-231 cell-derived exosomes (HELA-Exos) [146]. HELA-Exos showed strong anticancer activity in a mouse model and organoid model of human breast cancer. Here, the activation of natural DCs present in cDC1s in situ, thus improving the subsequent tumour-reactive CD8 + T cell responses. Immunofluorescence and flow cytometry experiments revealed that the infiltration of CD141 + natural DCs and CD8 + T cells along with the production of perforin and granzyme B in CD8 + T cells is significantly increased in HELA-Exo-treated tumours. A nanodrug delivery system containing DOX loaded in a fused exosome (prepared from DC and induced pluripotent stem cells) could effectively inhibit the progression of gastric cancer by the dual activity of chemotherapy and immunotherapy through the action of PD-1 antibodies [147]. Here, the effect of DOX chemotherapy (DOX@aiPS-DCexo) is synergized by releasing the suppressed T lymphocytes due to the action of modified PD-1 antibody from iPSCs. In PDT, for more stable and active delivery of photosensitizers, the exosomes are now used [148]. M1-Exos (exosomes isolated from M1 macrophages) can carry miRNA specifically to downregulate the expression of PD-L1 in gastric cancer cells [149]. Natural Killer cell-derived exosomes (NK-Exos) are found to contain cytotoxic proteins perforin, granzyme, etc. which in a co-culture of exosomes and tumour cells, could activate various caspases (3, 7, and 9) to induce tumour cell apoptosis without the addition of any external drug [150].

## Graphene and carbon quantum dots

Graphene, one of the crystalline forms of carbon, is formed from a monolayer of carbon atoms which tightly packed into a 2D honeycomb lattice. Graphene has various physicochemical properties along with a large surface area (> 2500 m^2^/g), distinctive optical behavioUrs, and excellent chemical stability [151]. Graphene sheets can be converted into graphene‐related materials like graphene oxide (GO), through oxidation and exfoliation of graphite‐bearing oxygen functional groups like hydroxyl (–OH), epoxy (–O), or carboxyl (C–OOH) on their edges and basal planes using the modified Hummers’ method. Graphene quantum dots (QDs) are semiconductor particles made from periodic table elements in groups IV–VI. These semiconductor nanocrystals have the potential to be employed as drug delivery vehicles or as fluorescent markers for tracking drugs during drug-tissue/cell interaction [152, 153]. Different staining patterns develop when QDs are utilized in the immunofluorescence staining of mortal against the proteins of hsp70 family, between normal and cancerous cells. A fluoro-immunoassay for detecting prostate-explicit antigens was created using streptavidin-covered QDs. The ability of carbon and graphene QDs (GQDs) to concentrate in a single internal organ allows targeted delivery, for bioimaging, stem cell delivery, biosensing, targeted gene delivery, and for PDT. Due to their distinctive 2-D structure, which offers a significant surface area for pi-pi stacking, graphene, and its variants have garnered a great deal of interest. Carbon QDs(CQDs) are another type of QDs with superior optical and biocompatibility properties which is useful for bio-sensing and imaging properties. To specifically target tumour cells, a compound of a CQDs-DOX was developed with increased retention inside tumour cells but reduced cytotoxicity than DOX alone [154]. Anticancer delivery of curcumin was improved in the presence of GQDs containing chitosan –PEG nanoconjugate (GQD-CS-PEG) against breast and colon tumours [155]. Using a mucin1 receptor-targeted aptamer along with curcumin it was observed that the anticancer effect of curcumin was more for mucin 1 overexpressing tumour cells with GQD-CS-PEG nanoconjugate.

Hyaluronic acid (HA)-loaded GQD delivery was found to be very useful for anticancer delivery. In one study, the redox-active ferrocene (Fc) containing Fc-GQD-HA, a biocompatible formulation, was found to target specifically CD44 over-expressing cancer cells with ~ threefold oxidative-stress-mediated Hela cell death [155]. The biocompatible GQD-HA-curcumin nanocomposite was found to show a high anticancer effect due to the presence of GQD as a drug carrier [156]. Further, the intrinsic fluorescence of GQD makes them visible during cellular entry making them dual purpose delivery system with targeted chemotherapy and bioimaging. The combined chemotherapy and PDT were observed with carbon QD and 5-aminolevulinic acid (5-ALA)-conjugated cyclodextrin-based delivery of DOX [157]. Laser-induced PDT was successful against MCF-7 cancer cells for these NPs with ~ 200 nm in size. The tumour-targeting peptide-conjugated GQD-based delivery of enzalutamide (TP-GQD-Enz) had shown better efficacy, both, in vitro and in vivo, against prostate cancer cells, [158]. The toxicity of GQDs is generally more dependent on size as < 20 nm size shows > 70% viability, whereas for > 60 nm size the viability decreases to < 25% on various cell types which are treated with 100 µg/ml of GQDs for 24 h [159]. Interestingly, when GQDs functionalized with different chemical groups like –COOH, –NH_2_, or –CO–N(CH_3_) were delivered, in vivo, does not show any significant difference in cytotoxicity [160].

## Carbon nanotubes

CNTs which are allotropes of carbon, were first discovered in the late 1980s. CNTs are completely composed of sp^2^ carbons and are from the members of the fullerene (hollow carbon allotropes with various shapes) structural family categorized as multi-walled CNTs (MWCNTs, 2–100 nm) or single-walled CNTs(SWCNTs, 0.4–2 nm). SWNTs and MWCNTs can naturally align themselves into cylindrical forms with diverse radiuses and chiral angles utilizing van der Waals forces. Because of these multiple alignments (sometimes even a million times), CNTs have a unique structure, and chemical and physical properties, including high mechanical strength, low density, and excellent conductivity. SWNTs are made up of monolithic cylindrical graphene, while MWNTs are made up of concentric graphene sheets [161]. CNTs are rolled-up tubular shells of the graphene sheet, a one-atom-thick layer formed of benzene-type hexagonal carbon atoms with lengths ranging from 1 to 100 nm [162]. The solubility of paclitaxel is improved in combination with cremophor which is toxic. Further, the formulation has a low circulation time which inspired PEG-functionalized paclitaxel for better circulation efficiency as compared to Taxol. But PEGylated SWNTs with the help of an ester linker, surprisingly increased the antitumour efficacy with high circulation time in comparison with both Taxol and PEGylated paclitaxel in the in vivo study of breast cancer [163, 164]. Folic acid conjugated with CNT and raloxifene (most commonly used to prevent and treat osteoporosis in postmenopausal women) binds to folate receptors and allows the nano-formulation to be phagocytosized by specific cancer cells. Targeted drug delivery enhanced the efficacy of raloxifene, which showed IC_50_ at 43.5 μg/ml [165]. These results suggest that carbon nanotubes can be a suitable option for anticancer drug delivery. In Table 4a summary was given for different NPs including GQDs, CQDs, CNTs, upconverting NPs (UCNP), and protein NPS (PrNPs) with possible mechanisms of action. Toxicity from SWCNTs was observed more than the MWCNTs as SWCNTs impaired the phagocytosis of alveolar macrophages at a very low dose of 0.38 mg/cm^2^, whereas for MWCNTs the same effect was observed with > ninefold dose [166]. The toxicity of CNTs is also observed with increasing length. For example, 825-nm-long CNTs produced higher inflammation and cytotoxicity than that induced by short-length CNTs (220 nm), in vivo, because macrophages cannot engulf the > 800-nm CNTs properly [167]. The accumulation of CNTs in different organs and their toxicity is also dependent on the functionalization of CNTs with various chemicals. As an example, MWCNTs functionalized with taurine accumulated in the liver for 3 months although diethylentriaminepentaacetic (DTPA)-functionalized SWCNTs of similar size were rapidly cleared from systemic blood circulation within 3 h with no deposition or toxicity [168].

**Table 4:** A summary of the applications of new generation NPs including GQDs, CQDs, CNTs, UCNPs, and PrNPs-based drug delivery against cancers and their possible mechanism of actions

Type of NP	Composition/Drugs	Study model	Used for cancer	Molecular Mechanism	Ref
GQDs	Chitosan –PEG nanoconjugate	MCF-7	Breast and Colon tumours	Selectively binds to the mucin-1 receptor	[155]
	Hyaluronic acid (HA)- loaded GQD	HeLa and L929 cells	Cervical cancer	Targeting a ligand with a high affinity for cell surface receptors as a target	[156]
	Targeting peptide- conjugated GQD enzalutamide (TP-GQD-Enz)	C4-2B or LNCaP cells Mice Model	Prostate cancer	Through a redox-sensitive mechanism, Enzyme is released under control at the location of the tumour	[161]
CQDs	5-aminolevulinic acid (5-ALA) conjugated cyclodextrin	MCF-7, WS-1	Breast Cancer	Inhibiting DOX will increase the system's therapeutic and synergistic benefits for breast cancer cells, and 5-ALA will produce ROS to have an irradiation effect on cancer cells	[157]
CNTs	PEGylated SWNTs with ester linker	4T1 murine breast cancer cell line	Breast Cancer	SWNTs functionalized with antibodies or peptide targeting ligands have demonstrated increased efficacy, in vivo	[164]
		Mice Model			
	Folic acid conjugated with CNT and raloxifene	MCF-7	Breast Cancer	Permits some cancer cells to phagocytose the nano-formulation by binding to the folate receptor	[168]
UCNP	Lanthanide-doped upconverting nanoparticles	HeLa cervical cancer cells	Cervical cancer	Production of singlet oxygen and other ROS after light excitation	[169]
	MC540/ZnPc-UCNP@Au	prostate cancer cells	Prostate cancer	ROS can be created through a photochemical process	[170]
	Rose Bengal-Modified Upconverting Nanoparticles	C6 glioma cells	Glioblastoma	ROS can be generated through a photosensitizer	[171]
	Combination of albumin with paclitaxel (Abraxane®)	Engineered zein protein nanoparticle	Metastatic breast cancer	Inhibition of cell division by increasing microtubule formation and stability, causing disruption of the microtubular network necessary for tumour cells to operate properly during interphase and mitosis	[172, 173]
	DOX-loaded albumin NPs [N-succinimidyl 3-(2-pyridyldithio) propionate (SPDP)]	Human Patients		By enhancing permeability and retention (EPR) impact, nanoparticles enable passive tumour targeting and solve the issues with multidrug resistance	
PrNPs	STAT3i SPNPs	MCF-7 and MDA-MB-231 HepG2 cells and L929 cells GL26 mouse GBM and HF2303 human GBM stem cells	Breast Cancer Hepatocellular carcinoma and murine fibroblast Glioblastoma	Production of ROS was linked to mitochondrial damage and apoptosis. PDT boosts the susceptibility of tumour cells to chemotherapeutic agents by inhibiting a number of mediators of multidrug resistance Hypericin-based photodynamic therapy for hepatocellular cancer Mechanism causing its tissue penetration abilities	[174] [175] [176]

## Upconverting nanoparticles

**Fig. 5:**
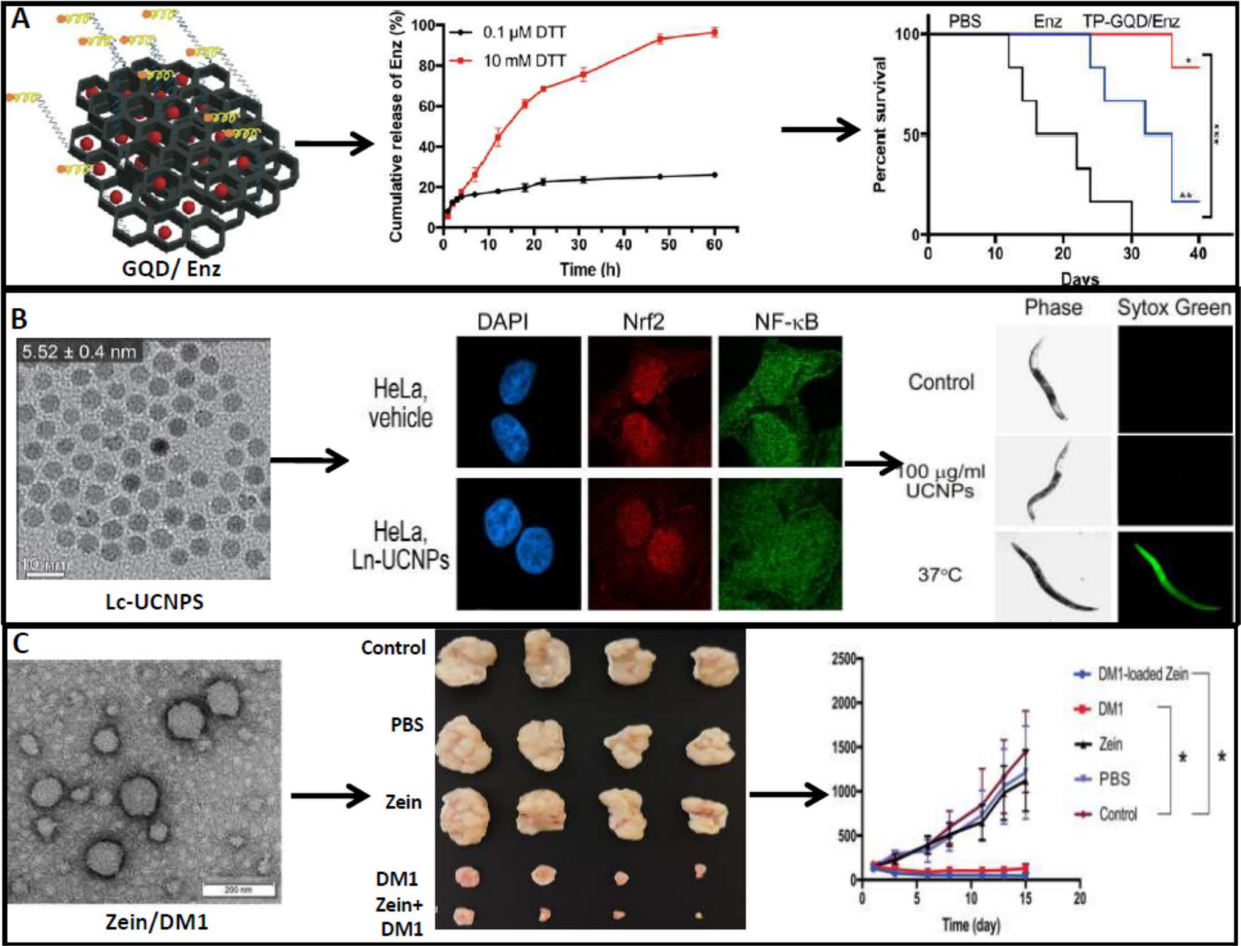
Graphene quantum dot, upconverting, and protein NP-based drug delivery for cancer. **A** The disulphide cross-linked GQD composed of tumour targeting peptide and Enzalutamide (Enz) as drug shows DTT-responsive increased drug release and better survival of mice model of prostate cancer in intravenous delivery. **B** Lanthanide-doped UCNPs (Lc-UCNPs) do not cause any change in Nrf2 and NF-κβ expression and toxicity as measured in *C. elegans* fluorescence in Hela cancer cells. **C** Protein NP composed of Zein and Maytansine (DM1) (112 nm) shows significant reduction of tumour in A549 tumour-bearing nude mice. The **A** and **C** were adapted from references [161] and [185] for which copyright permission is not required. The **B** was reproduced/adapted from reference [169] with permissions from [American Chemical Society], copy rights reserved.

Upconverting nanoparticles (UCNPs) are nanoscale particles (diameter 1–100 nm) that can convert two or more incident photons of relatively low energy into one emitted photon with higher energy photons [177]. UCNPs are crystalline nanomaterials made from inorganic materials that can convert NIR excitation light into UV–visible emission range. Due to excitation with NIR, there is minimum background autofluorescence and reduced light scattering, allowing deeper penetration into biological samples. Due to this upconversion property where generally, absorption occurs in the IR, while emission occurs in the visible or UV region, the UCNPs gained a lot of interest in PDT-based therapy [178]. Tumour cells and sub-cellular organelles, tumour microvasculature, and the inflammatory and immune host system are all targets of PDT where UCNPs can be delivered using different photosensitizers (PS) [169, 179]. Under NIR light stimulation, UCNPs can emit high-energy visible light, which can activate surrounding PS molecules, causing singlet oxygen to be produced, thereby accumulating a high amount of ROS inside cancer cells to kill them [170]. The UCNPs prepared by high-temperature co-precipitation or surface modification by silica or PEG make them resistant to non-specific binding. Further, the conjugation of streptavidin or antibodies allows biological detection [180]. When applied for immunocytochemistry analysis, UCNPs are > 50-fold better than conventional fluorescent labels for the detection of breast cancer marker EGFR2. UCNPs of 26 nm were synthesized from lanthanides by a high-temperature coprecipitation method. The particles were modified by PEG and Rose Bengal (RB) as PS and RB-conjugated UCNPs can selectively kill rat glioma cell lines (C6) due to the production of high ROS when irradiated at 980 nm [171]. In another study, the UCNPs are conjugated with EGFR-antibody and enzyme cytosine deaminase (CyD). The EGFR antibody allows specific targeting of the NPs inside the tumour cells followed by conversion of the prodrug 5-fluorocytosine (5-FC) to the anticancer drug 5-FU by the enzyme CyD. This prodrug-based antibody-conjugated UCNPs when irradiated with NIR light yielded twofold slower growth of tumours than control groups when 5-FC was administered orally in mice model [181]. Graphene quantum dot, upconverting, and protein NP-based drug delivery for cancers are shown in Fig. 5 which includes published data with permissions. The bioavailability and toxicity of lanthanide-doped UCNPs were measured after oral and intravenous administration routes, in vivo. It was observed that there is no observed toxicity even with a high dose of 100 mg/kg body weight [182]. When polyacrylic acid (PAA) and PEG-coated UCNPs (NaYF_4_:Tm^3+^,Yb^3+^) were delivered at a dose of 20 mg/kg, there was no organ damage or lesion in mice although aggregates of non-luminescent UCNPs were found in the liver after 7 days due to degradation of UCNPs by the macrophages [183]. Among different UCNPs the gadolinium-based NPs are found to be more toxic due to the leaching of Gd3 + ions from the complex causing possible nephrotoxicity [184].

## Protein nanoparticles

**Fig. 6:**
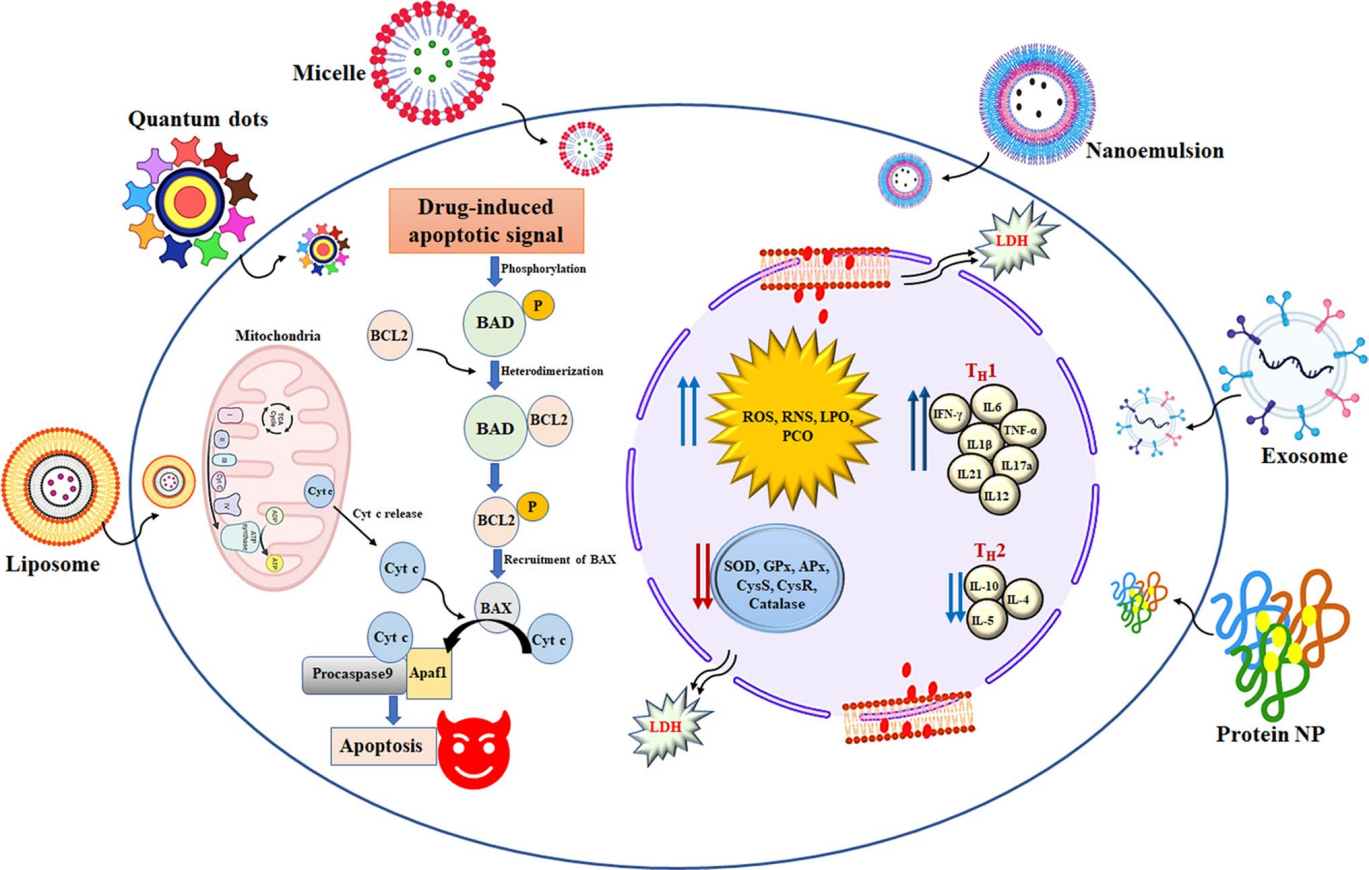
Mechanistic evaluation of NPs-based drug delivery for cancer. Reactive oxygen species, reactive nitrogen species, lipid peroxidation, and protein carbonylation are major oxidative stress which leads to cytochrome c-mediated apoptosis and Th1/Th2 cytokine imbalance against cancer cell

High biocompatibility and ease in synthesis allowed the synthesis of PrNPs from various proteins including fibroins, albumin, gelatin, lipoprotein, gliadine, ferritin, etc. which have compact 3-D structures to functionalize various drugs/molecules [186]. When the proteins are in the form of NPs they are less immunogenic than normal proteins. In cancer therapy, the first protein NP (PrNPs) which was approved by FDA for clinical use is Abraxane® which is a combination of albumin with paclitaxel for the treatment of metastatic breast cancer. This formulation is synthesized from human serum albumin which evades side effects like hypersensitivity which was the problem associated with the previous paclitaxel formulation Cremopho [172, 173]. Numerous functional groups associated with amino acid side chains of PrNPs help in various conjugation of drugs or ligands easily by click chemistry [172]. The high surface volume ratio of these PrNPs allows very high drug loading [172]. DOX-loaded albumin NPs were synthesized with the modification of N-succinimidyl 3-(2-pyridyldithio) propionate (SPDP) for reducing environment-based drug release. These PrNPs of ~ 315 nm showed the potential anticancer effect on the breast cancer cell lines—MCF-7 and MDA-MB-231. The toxicity of this formulation was negligible as it tested on the non-cancer cell MCF10A [174]. Zein is a high proline-rich protein obtained from maize. The delivery of maytansine (DM1), a potent anticancer drug, whose use is limited due to its poor water solubility and toxic side effects, with zein NPs exhibits a better antitumour efficacy than DM1 where tumour inhibition rate was 97.3% and 92.7%, respectively [185, 187]. Poor water-soluble drugs like luteolin, a flavonoid, loaded with zein NPs (size ~ 250 nm) increased the bioavailability with the advantage of oral administration of luteolin. It kills the cancer cells by arresting the cell cycle followed by apoptosis [188]. In another study, an engineered zein protein nanoparticle was used for hypericin-based photodynamic therapy for hepatocellular cancer (HepG2) cells [175]. Using polymerized human serum albumin, a synthetic PrNP (SPNP) was prepared which contains siRNA against Signal Transducer and Activation of Transcription 3 factor (STAT3i) and equipped with the cell-penetrating peptide iRGD [176]. The delivery of STAT3i SPNPs, compared to ionized radiation-based standard care, showed long-term survival in ~ 90% of glioblastoma-bearing mice with high immunological memory [176]. In Fig. 6, the major mechanisms involving different NP-mediated cell death against cancer cell are summarized.

## Nanovaccines for cancer immunotherapy

It is expected that the way vaccination worked for viruses or bacteria, where inactivated whole cell lysates were administered with adjuvants, the same principle should work for cancer immunotherapy. However, clinical trials studies containing tumour lysate (TL), for example Melacine (an allogenic melanoma TL vaccine) when administered with a DETOX adjuvant, resulted in < 8% positive clinical responses [189]. Using the cancer cell membrane fractions (CCMF) from TLs, Jin et al.,[182] coated PLGA NPs which showed increased NP trafficking to lymph nodes (LNs). The CCMF-PLGA NPs coined as “artificial cancer cells” showed > 30% inhibited migration of tumour cells towards human mammary fibroblasts in vitro and tumour protection, in vivo, in murine lung metastasis model after day 21 postinoculation subcutaneously. This happened due to targeted activation of cytotoxic T lymphocytes (CTLs) because these are accumulated in LNs where IFN-γ-producing CD4 + and CD8 + T cells were abundant. Apart from the TL-based studies, immunogenic cell death (ICD) of tumour cells, which also serve as a rich source of tumour antigens and danger signals, was used for improved vaccination strategy. In a study using multilamellar liposomes with (diameters ~ 250 nm) maleimide-functionalized different lipids were synthesized by incubation with thiolated hyaluronic acid and CpG (cytosine–guanine dinucleotide) motif [190]. This led to the formation of homogeneous NP and increased stability in vivo. When ICD was induced in B16F10-OVA tumour cells maleimide-displaying CpG nanodepots (Mit-B 16F10-OVA-CpG-NPs) significantly upregulated the costimulatory molecules CD40 and CD86 as along with inflammatory cytokines (IL-12p70, IFN-β, TNF-α) which are associated with increased antigen presentation and T cell activation. More importantly, mice vaccinated subcutaneously with Mit-B16F10-OVA-CpG-NPs resulted a > 2.4-fold increase in antigen-specific CD8 + T cell responses, than the control. Mice challenged with B16F10-OVA cells after 8 days of postvaccination with Mit-B16F10-OVA-CpG-NP showed 100% rejection of tumour cells compared with 20% for the Mit-B16F10-OVA.

**Fig. 7:**
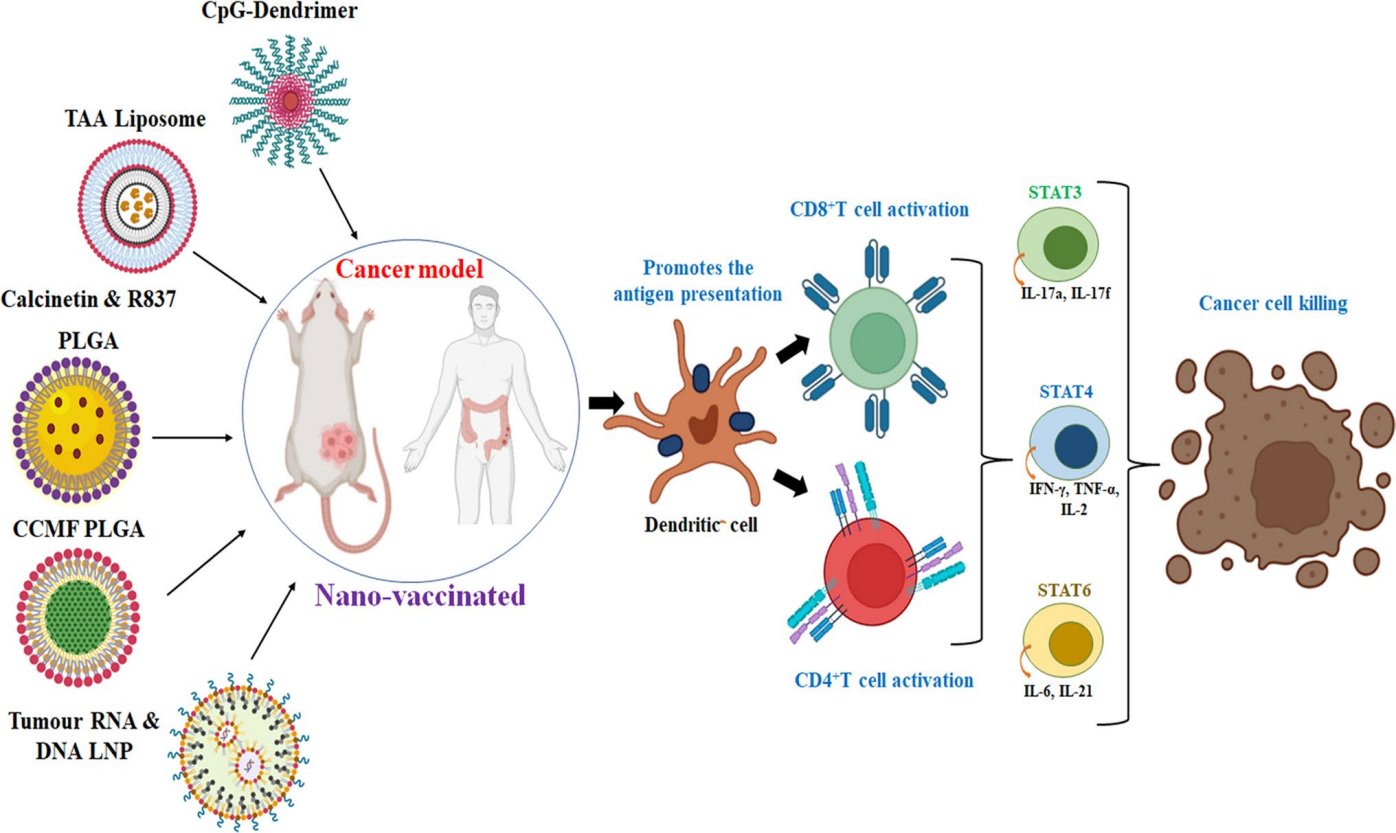
Nano-vaccine candidates with adjuvant enhance the immune response by the dendritic cells-based increased antigen presentation. This further stimulates the CD8 + T and CD4 + T cells by triggering the various signal transducer and activator of transcription (STAT) signalling, thereby releasing various cytokines (IFN-γ, TNF-α, IL-2, IL17a, IL-6, and IL-21) leading to tumour cell death by autophagy, apoptosis, or CTL-mediated death

Tumour-associated antigens (TAAs) and tumour-specific antigens (neoantigens), which are mutated, non-self-proteins derived from tumour cells are two major types of tumour antigens. Since neoantigens are non-self, most antigenic neoantigen-based vaccines are expected to avoid central immune tolerance and induce tumour-specific T cell activation without safety concerns [191]. Nanodiscs prepared with sHDL (synthetic high-density lipoproteins) by hydrophobic interactions incorporation of neoantigen-lipid conjugates and CpG (a Toll-like receptor-9 agonist) are efficiently taken up by dendritic cells (DCs), leading to strong colocalization with endosomes/lysosomes which are sustained high level of epitope-MHC-I presentation, and CD8 + T cells [192]. Calcinetin-expressed cancer cell membrane antigen was coated on a nanovaccine composed of PLGA polymer and adjuvant R837[193]. The nanovaccine delivers the adjuvant R837 and calcinetin membrane antigen, together, triggering a personalized immune response to the corresponding tumour and inducing the active uptake of DCs with a long-lasting memory. The antigen ovalbumin and the adjuvant CpG were loaded with polycationic PEI by electrostatic binding to deliver as nano-vaccine [194]. When the nanovaccine was delivered in combination with the enzyme hyaluronidase, more accumulation of the nanomaterial was observed inside the tumour cells due to the breakdown of hyaluronic acid present on tumour extracellular matrix and consequent uptake of nanovaccine leading to more activation of DCs. Apart from tumour antigens/membrane fractions, the nucleic acid present in the tumour cells can be used for a nano-vaccination strategy as well. In this study, total tumour RNA isolated from liver cancer cells (Hepa1-6) were loaded with liposome-polycation-DNA (LPD) complex NP by thin-film hydration method [195]. The RNA-LNP nanovaccine (size ~ 205 nm) is capable of inducing DC maturation and inducing CTLs to kill Hepa1-6 cells, in vitro and, further, prevent hepatocellular carcinoma growth; in vivo. Various NPs-based vaccine development with possible mechanisms of immune response involving CD8 + and CD4 + T cells is depicted in Fig. 7.

## Advantages of cancer immunotherapeutics through nanoscale delivery

In modern cancer immunotherapy, immune checkpoint inhibitors (ICIs) have gained the maximum attention. The checkpoint proteins like program death (PD)-1 (present in T cells) bind with PD ligand-1 (PD-L1) which are, generally, overexpressed in most cancer cells. This binding acts as an ‘off-signal’ for immune-mediated signalling so that cancer cell can evade the T-cell-mediated death. The cytotoxic T-lymphocyte-associated antigen 4 (CTLA-4) and lymphocyte activation gene-3 (LAG-3) are similar kind of checkpoint proteins on immune cells which are used by cancer cells to bind and evade the immune cell signalling-mediated death. The ICI-targeted drug delivery is so important that US FDA has successfully approved 3 different categories of ICIs such as PD-1 inhibitors (Pembrolizumab, Nivolumab, and Cemiplimab), PD-L1 inhibitors (Durvalumab, Atezolizumab, and Avelumab), and CTLA-4 inhibitor (Ipilimumab) in last 13 years. These are humanized monoclonal antibodies (MAB) which are in use for in patients with metastatic melanoma, non-small lung cancer, head & neck cancers, and renal cell carcinoma. Due to high cost of MAB treatment and its side effect NP-based delivery with siRNA for inhibition of ICIs was tried for cancer therapy. A liposomal co-delivery of PD-L1 small interfering RNA (siRNA) and anemoside B4 (a saponin having anti-inflammatory activity) was evaluated for anticancer efficiency in mouse models of LLC and 4T1 tumours. The liposome (AB4/siPD-L1, particle size ~ 180 nm) showed high drug encapsulation [196], high stability in serum and pH-responsive release properties. Liposomal delivery showed the antitumour T-cell response with long-term memory by downregulating the PD-L1 expression and increased T cell response. In another study, co-delivery of DOX and PD-L1 siRNA was done against breast cancer cells with liposomal pH-sensitive delivery system capable of escaping the endosomal degradation [197]. The high accumulation of siRNA at the tumour site was confirmed by in vivo imaging, which is associated with downregulation of PD-L1 expression and tumour cell death.

A ROS-responsive NP was prepared with a heptapeptide (T7) which was used to co-deliver DOX and PD-L1 siRNA against 4T1 tumour bearing mice, in vivo. The T7 element allows NP binding on the tranferrin receptor of cancer cells with increased apoptosis under ROS followed by improved delivery of DOX and siRNA leading to downregulation of PD-L1 and tumour cell death [198]. Zhang et al. [199] created a nanovesicle (NV) from the membrane of transfected HEK 293 T cells to load the following components –PD-L1 receptor tagged with DsRed protein, dendritic cells (DC) and 1-methyl tryptophan, an inhibitor of indolamine 2,3-dioxygenase (IDO), which is an immunosuppressive molecule overexpressed by tumour. The NV encapsulated with PD-L1 + IDO + DC provides immune tolerance in TME along with the increased density of CD8 + tumour infiltrating lymphocytes which reduced the tumour size in mice drastically. All of these studies proved that antitumour responses can be enhanced by disrupting the PD-1/PD-L1 immune inhibitory axis through NP-based delivery of PD-1/PD-L1 molecule or its siRNA.

In an interesting study the mRNA coding for Pembrolizumab was delivered intravenously through a lipid-based NP (LNP-Pembrolizumab). The serum levels of produced antibody were tested both in vivo and in vitro which showed a dose-dependent increase (in C57Bl/6 mice the antibody concentration was > 20 µg/ml with 0.5 mg/kg dose of LNP-Pembrolizumab [200]. Pharmacokinetics studies showed at least 2.5-fold higher accumulation of serum antibody from LNP-Pembrolizumab (dose of 2.0 mg/kg) treatment than standard Pembrolizumab antibody (dose of 5.0 mg/kg) with a consistently higher area under the curve till 35 days (AUC_0-35 days_). This LNP-based & protein-free delivery of Pembrolizumab through intravenous mRNA delivery reduces the cost of delivery and clinical complicacies of monoclonal antibodies.

Apart from ICI-based immunotherapy, the NP-based delivery with evasion of cancer tumour microenvironment can also provide better immunotherapy where stimuli-responsive drug delivery was useful. Wang et al. constructed an amphiphilic pH-responsive galactosyl dextran-retinal (GDR) nanogel for cancer vaccine delivery where dextran was conjugated through a pH-sensitive hydrazone bond with all-trans retinal (a metabolite of vitamin A). This was further modified by galactosylation to acquire dendritic cell (DC)-targeting ability. The pH-sensitive NP-triggered lysosome rupture at low pH could directly induce ROS production in DCs, leading to enhanced proteasome activity and downstream MHC I antigen presentation [201]. A pH-responsive biodegradable poly (ε-caprolactone)-block-poly (ethylene glycol) (PCL-b-PEG) vesicles were prepared encapsulating tumour endogenous antigens HSP-70 chaperoned peptides (HCP) and adjuvants CpG ODN. Using two strong antigens they could efficiently activate APCs and then trigger CTL and long-term memory immune responses. The NPs were also loaded with CD80 ab so that it can bind with the receptor on DCs. The drug release TME-responsive since > fivefold drug was released at pH 5 than in pH 7.4. The intra-tumoural immunity in 4T1 tumour bearing mice was measured based on increased release of CD4 + and CD8 + T cells. HCP-CpG-CD80ab containing NPs completely reduced the tumour growth to normal in 27 days, and the tumour growth is completely inhibited even after 32 days of tumour re-challenge indicating the presence of strong memory in NP-treated animals [202].

## Nanotechnology for the detection of cancer biomarkers

Early detection, rather than therapy, is possibly more suitable for cancer prevention since the later stage of cancer is generally more expensive to treat and, more often, untreatable. Liquid biopsy, which is more minimally invasive than conventional tissue biopsy, allows the detection of a large variety of circulating biomarkers, such as microRNA (miRNA), cancer-specific antigens/proteins, circulating tumour DNA (ctDNA), circulating tumour cells (CTCs), exosomes, mRNA and associated genetic mutations, etc. Over a long period clinical detection of cancer primarily relies on imaging techniques (X-ray, CT, MRI, endoscopy, ultrasound, etc.) or the histopathology analysis of the suspected tissue which is limited in their ability to differentiate between benign vs. malignant lesions, sensitivity, cost-effectiveness, and poor patient compliance. Nanotechnology-based detection which is generally comprised of optical, fluorescent, or electrochemical sensors, can detect a vast range of potential cancer biomarkers even in picomolar (pM) quantity with limited cross-reactivity from suspected patient’s blood, serum, cough, urine, etc.

Hairpin DNA complementary to the tyrosinase mRNA was conjugated with GNPs for the successful detection of the overexpressed tyrosinase gene in melanoma cells [131]. Mini chromosome maintenance protein 5, MCM5, which overexpressed protein in cervical cancer, was detected at a sensitivity of < 3 pM using a GNP-based electrode which is immobilized with capture antibody against MCM5[203, 204]. A new electrochemical immunosensor is developed to simultaneously detect 3 early breast cancer biomarker antigens mucin1 (MUC1), cancer antigen 15–3 (CA15-3), and human epidermal growth factor receptor 2 (HER2) with a detection limit of < 2 ng/ml which is significantly lower than the clinically relevant cut-off values [205]. Here the polyethyleneimine-coated gold nanoparticles (PEI-GNP) was conjugated with different capture antibodies (specific for 3 antigens) and deposited with three different redox system for the detection of all three antigens together. The prostate-specific antigen (PSA), a glycoprotein of 237 amino acids that are released by the prostate gland, is the most important and common biomarker for prostate cancer. A biosensor based on Zn(II) metal–organic framework NPs can detect the PSA at a detection limit of 0.145 fg/mL where the PSA concentration > 10 ng/mL is considered a threat for suspected prostate cancers [206]. The antibodies against PSA and prostate-specific membrane antigen (PSMA) were decorated on Fe_3_O_4_ nanoparticles which are loaded onto graphene oxide (GO) nano-sheets for microfluidic immunoarray based on electrochemical reaction [207]. The detection limit was 15 fg/mL for PSA and 4.8 fg/mL for PSMA from human serum which is > 1000-fold better than the earlier report of PSA detection using Fe_3_O_4_.

An antibody-free PSA detector, which is of low-cost electrochemical biosensor, was developed using GNP-MWCNT (multi-walled carbon nanotubes)-based aptasensor with a lower detection limit of 1 pg/ml [208]. A new lung cancer biomarker, GM2 activator protein (GM2AP), was detected with a detection limit of < 1 pg/ml from human serum and urine samples using a PEI-GNP and phosphomolybdic acid (PMA) modified electrode [209].

**Fig. 8:**
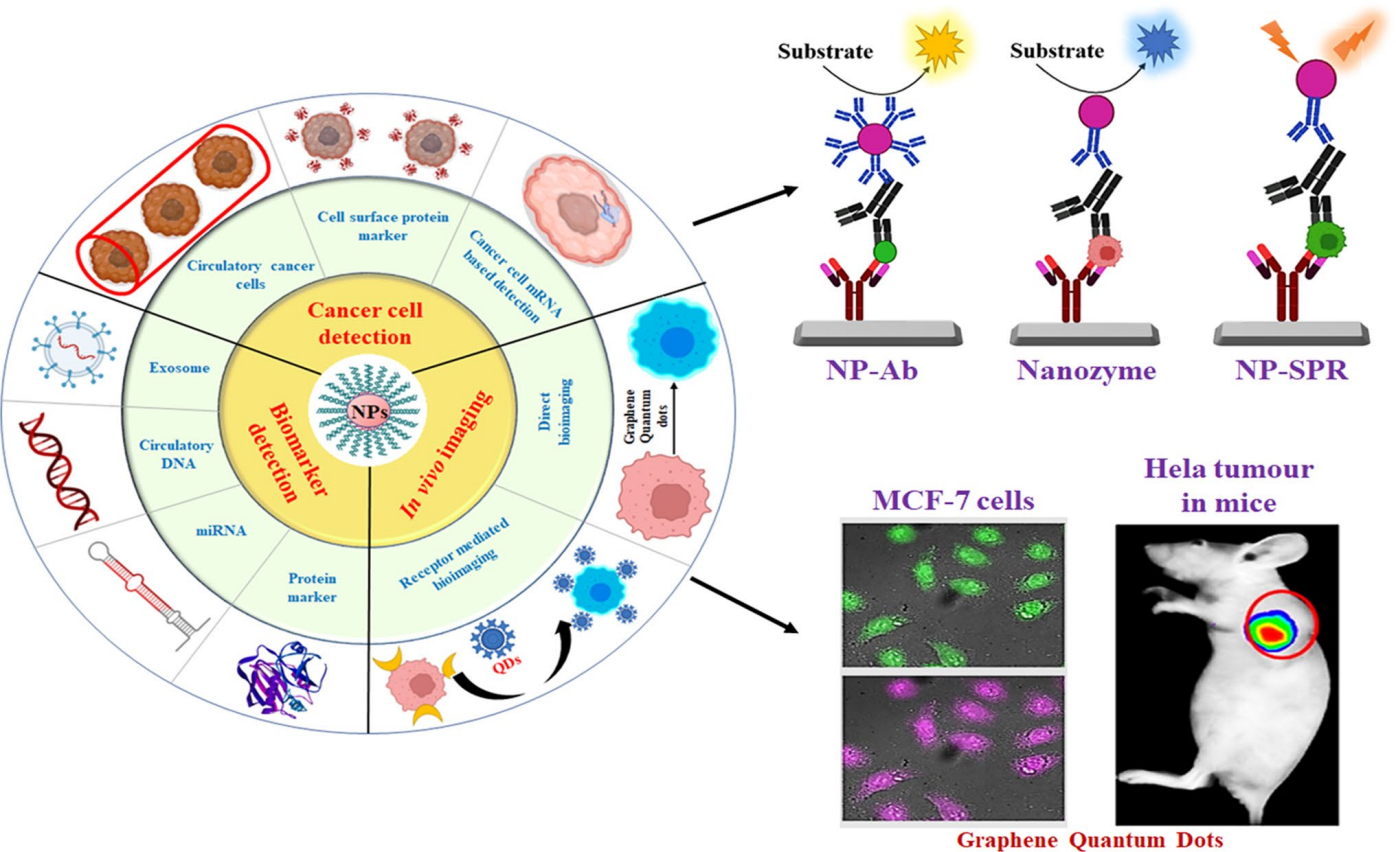
Nanoparticles-based cancer detection. Different types of cancer markers (antigen, miRNA, circulatory cells, DNA, mRNA, exosomes, etc.) can be detected using NP-antibody, Nanozyme, or NP-based surface plasmon resonance phenomenon (SPR). Quantum dots are also used for fluorescent-based bioimaging for in vitro (MCF-7 cells) and in vivo tumour models

High overexpression of proteolytic enzymes matrix metalloproteinase (MMPs) is associated with malignancies. A dual detection system of the colorimetric and fluorimetric platform was enabled by green emitting gold nanoclusters which were synthesized using gelatin as substrate [210]. The semi-quantitative detection of MMP-9 enzyme by the naked eye using gelatinase assay provides a limit of detection (LOD) of 2 ng/ml and 0.25 ng/ml for colorimetric and fluorimetric methods, respectively. Apart from the detection of cancer antigens and enzymes there are several nano-based platforms for the detection of miRNA/nucleic acids from cancer cells. Upregulated miRNA targets, which control gene expression and important biomarkers for cancers, like miR21 and miR221 from colorectal cancer patient plasma were detected using bimetallic nanostar using the principle of surfaced-enhanced Raman scattering (SERS) attached with gene probes called the inverse Molecular Sentinel (iMS). The resulting SERS data with a LOD of 6.8 and 16.7zmol for miR21 and miR221, respectively, correlated well with PCR data with a clear distinction between patients and healthy samples [211]. In an interesting study, using GNP-based dual cycling nanoprobes (DCNPs) detection of tumour exosomes, exosomal protein, and miRNA was made possible [212]. The LOD for tumour exosomes, exosomal protein (ExoPD-L1), and exosomal miRNA (ExomiR-21) was 10 particles/μL, 0.17 pg/mL, and 66 fM, respectively. Such a DCNP-based strategy allows simultaneous changes of exosomal protein and miRNA expression together, which are generally regulated by signalling molecules or therapeutic delivery. In Fig. 8, we summarize different strategies for NP-based biomarker detection of cancers using antibodies, nanozymes, SPR, etc. along with bioimaging.

## Approved cancer nanomedicines and the future of clinical trials

USFDA and its equivalent in the European Union (EU), the European Medicines Agency (EMA), have approved > 250 nanomedicine-based drugs for diagnostic and therapeutic purposes of various human cancers, and many other formulations are currently being evaluated. Liposomes are the best delivery system so far as many liposomal formulations are in clinical trial and some are FDA-approved. In fact, Doxil, approved by FDA, is the first nanomedicine that is PEGylated liposomal version of DOX for the treatment of ovarian cancer and multiple myeloma. After that DaunoXome (liposomal DOX) and Myocet (non-PEGylated liposomal DOX) were approved for HIV-related Kaposi sarcoma and metastatic breast cancer in the year 1996 and 2002, respectively [213]. During the year 2009 to 2017, various liposomal formulations Mepact (Mifamurtide), Marqibo (Vincristine sulphate), Onivyde (Irinotecan), Vyxeos (Cytarabine) were approved for the treatment of various cancers including AML. Different lipid complexes and liposomal nanoformulations, including Abelcet, Amphotec, and Ambisome, for parasitic/fungal diseases were approved later by FDA after the success and popularity of Doxil [214]. The first paclitaxel nanoformulation for the treatment of different cancers known as Lipusu is approved in 2006 by the State FDA of China. The major reasons for the rapid development of liposomal formulations are the reduced toxicity and sustained release of the drug at the tumour sites. Polymeric NP-based nanomedicines Eligard (Leuprolide acetate) and Genexol-PM (paclitaxel) were approved in the year 2002 and 2006, respectively, for the treatment of advanced prostate cancer and NSCLC.

The first protein-based nanomedicine that was approved (Ontak-1999) is a recombinant DNA-derived cytotoxic protein that is composed of Il-2 and diphtheria toxin. Later, in the year 2005 paclitaxel as an albumin formulation known as Abraxane was approved for the treatment of NSCLC, breast, and pancreatic cancers [175]. FDA has now approved other proteins such as gelatin, zein, and milk proteins of β-lactoglobulin & casein for pharmacological applications which may trigger more clinical trials with protein-based NPs in the future [173]. PEGylated protein conjugate of L-asparaginase known as Oncaspar is approved by EMA in the year 2016 for the treatment of acute lymphoblastic leukaemia. The major reason for the success of these nanomedicines is due to targeted drug delivery with sustained drug release causing less toxicity. Sometimes, approved vaccines and drugs are delivered with nanocarriers for improved preclinical effect. Exemestane, an aromatase inhibitor that has been approved by the US FDA for the treatment of breast cancer, is limited in applications due to its poor aqueous solubility and low oral bioavailability. When exemestane is delivered with thymoquinone, a natural chemosensitizer and chemotherapeutic derived from the oil of the seeds of Nigella sativa Linn, the combinatorial delivery showed a high anticancer effect [215]. All FDA-approved anticancer drugs, which are immune checkpoint inhibitors (ICIs), are limited by the low response rates & adverse immune-related side effects in normal organs, due to their non-specific and systemic in vivo distribution. The nanocarrier-based delivery of these ICIs (namely Ipilimumab, Nivolumab, Atezolizumab, etc.) is now being examined for their improved anti-tumour effect with reduced side effects [216]. Inorganic and metallic NP-based agents are on the high rise for cancer imaging purposes but are not very common for therapeutic deliveries. A hafnium oxide NP, known as NBTXR3 (~ 50 nm), was approved for the radiotherapy of locally advanced soft-tissue sarcoma in 27 European countries during in 2019[217]. Due to radiosensitization, the emery of NTXR3 can be increased > 9 times of absorbed energy which enhances the targeted killing of the tumour by radiotherapy.

Cornell dots (nomenclature came from the discovery by a group from Cornell University, USA), prepared from biodegradable silica NPs and PEG, has been approved recently by the FDA as a targeted imaging agent for a stage I clinical trial [218]. The application of SPION, an iron oxide-based NP with polysaccharide coating, has revolutionized the applications of metallic NPs for high-resolution multimodal imaging. This is followed by the approval of other SPION-based contrast agents (Cliavist, Feridex, and Gastromark) for in vivo imaging [219]. Further, Feraheme and NanoTherm are modified SPIONs containing formulations that are approved as therapeutic agents for treating anaemia and glioblastoma, respectively [219]. Acticoat, an SNP-based wound dressing, was developed in 1998 followed by other SNP-based products (Bactigras, PolyMem Silver, and Tegaderm) for the same application. But till to date pure GNP and SNP-based formulations are not approved for systemic cancer therapy. In Table 5 and Table 6 the list of different NP-based drugs/formulations for various cancers is summarized which are approved and in different phases of clinical trial, respectively.

**Table 5:** FDA-approved nanomedicine products against various cancer

Product	Company	Indication	Refs.
Doxil	OrthoBiotech	Metastatic ovarian cancer	[214]
Abraxane	Albumin-bound paclitaxel particle	Lung cancer, breast cancer, others	[175]
Feridex IV, Gastromark Combidex (Ferumoxtran-10)	Advanced Magnetics	Enhanced MRI Contrast	[219]
NanoTherm	MagForce	Solid tumour	[220]
Myocet®	Zeneus Pharma Sopherion Therapeutics	Cardioprotective formulation of doxorubicin used in late-stage metastatic breast cancer	[214]
Oncaspar®	Sigma-Tau Pharma	Lymphoblastic leukaemia	[215]

**Table 6:** Different NPs-based formulations under different phases of clinical trial for various cancers

NPs	Drug name/active ingredients	Cancer type	Status	Refs.
Liposome	Paclitaxel liposome	Advanced Pancreatic Cancer	Phase 4	[221]
Liposome	Liposomal Doxorubicin	Desmoid tumour	Phase 3	[222]
Liposome	Liposomal bupivacaine	Ovarian Cancer	Phase 3	[223]
Liposome	Liposomal doxorubicin + Cyclophosphamide vs Docetaxel + Cyclophosphamide	Breast Cancer	Phase 4	[224]
Micelle	Paclitaxel	Advanced Solid tumours	Phase 2	[225]
Polymeric micelle	Docetaxel-PM	Oesophagus Squamous Cell Carcinoma (SCC) Metastatic Cancer	Phase 2	[226]
Iron oxide	Ferumoxytol	Liver Neoplasms	–	[227]
Nanoemulsion	5-aminolevulinic acid	Lentigo Maligna	Phase 4	[228]
Quantum dots	Quantum dots coated with veldoreotide	Breast Cancer Skin Cancer	Phase 1	[229]

Paclitaxel-liposome combined with S-1 (an orally administered fluorouracil) is in a phase 4 trial for the treatment of advanced pancreatic cancer. The nanoformulation can prolong the patients’ median progression-free survival (PFS) and median overall survival which were 6.50 months and 13.00 months, respectively, with tolerable toxicity [221]. In another study, a randomized, double-blind, placebo-controlled study is going on to compare differences in PFS in patients treated with liposome-DOX or placebo (liposome adriamycin 50 mg/m^2^), against desmoids tumours (often occur in the abdomen, arms, and legs showing non-cancerous growths) with 72 patients in phase III clinical trials in Guangdong, China [222]. The current research shows that DOX under the encapsulation of liposomes prolongs the half-life of the drug, and reduces the cardiotoxicity, with enrichment in the tumour sites to improve the anti-tumour activity.

In addition, liposomal DOX has better clinical perception since it causes less hair loss during chemotherapy, a common side effect [230]. Bupivacaine (discovered in 1957 and sold under the brand name Marcaine as a local anaesthetic) and liposomal bupivacaine with re-dosing at 48–60 h are being used in a non-randomized clinical trial of 45 patients undergoing major gynaecologic procedures to find out which type of transverses abdomens plane (TAP) block improves pain control and lowers the risk of postoperative common side effects of surgery [230]. Although the outcome is still awaited, the FDA has recently approved liposomal bupivacaine as a longer-acting form of the anaesthetic for larger applications.

A randomized phase 3 trial was evaluated with 422 patients to check whether lurbinectedin (a synthetic alkaloid analogue used for the treatment of small cell lung cancer) improved PFS compared to PEGylated liposomal DOX (PLD) or topotecan against ovarian cancer patients who are resistant against cisplatin [223]. However, the trial failed to give any selective advantage for PLD over lurbinectedin since the median PFS was 3.5 and 3.6 months for each group, respectively. There is a open-label, multicentre, non-inferiority, randomized controlled clinical study with 372 patients to evaluate the efficacy and safety of a PLD + cyclophosphamide followed by docetaxel plus trastuzumab and pertuzumab (PLD + C + HP followed by THP)[224]. The comparative arm is with a docetaxel + carboplatin along with trastuzumab and pertuzumab (TCbHP) regimen for HER-2-positive breast cancer patients with neoadjuvant treatment. The recruitment for this study is still going on with no updates on the outcome yet, but it is expected that PLD + C + HP + THP treatment will give a better indication than TCbHP with a lower cost of treatment. A Phase I Study with an estimated 98 patients was planned to evaluate the pharmacokinetics, safety, tolerability, and preliminary efficacy of injectable paclitaxel micelles for Chinese patients with advanced solid tumour [225]. The major purpose is to determine the maximum tolerated dose (MTD) and dose-limiting toxicity (DLT) of paclitaxel micelles for injection and explore phase II clinical dosages in the future with reduced side effects. Polymeric micelle-based Docetaxel is under phase 2 trial for oesophagus squamous cell cancer with 38 patients. It is well now established so far that this polymeric nanoformulation is non-toxic with an entire response rate of 34% and a median survival duration of 11.6 months [226]. Iron oxide (SPION)-based MRI cellular imaging (MRI-SPION) was planned to reduce radiation-induced liver disease after stereotactic body radiotherapy in HCC in a phase 1 trial with 28 patients [227]. Hepatic SPION accumulation will be quantified to see the effect of the MR-Linac-SPION-based quantitative treatment-planning platform during liver neoplasm. Lentigo maligna (LM) is an *in situ* form of melanoma that occurs on sun-exposed skin. Untreated LM can change into adverse invasive LM melanoma. Nanoemulsions of 5-aminolevulinic acid (Ameluz®) were used in combination with ablative fractional laser (AFL)-assisted PDT. In this non-sponsored prospective pilot study, ten patients with histologically verified LMs were diagnosed with AFL-assisted PDT three times at 2-week intervals using a light dose of 90 J/cm^2^ where the primary outcome showed histopathological clearance of the LM, completely, from the surgical specimen [228]. However, due to severe skin reactions and pain (for which local anaesthesia with ropivacaine was used) during surgery, it was suggested that AFL-assisted PDT + Ameluz® can only be used for inoperable conditions [231].

Quantum dots of CdS/ZnS that are decorated with veldoreotide and somatostatin (a peptide hormone) analogue, are used to serve the dual purpose of delivery of anticancer drugs and the bioimaging of breast cancer cells with very low immunogenicity [229]. A PLGA NP contains a tumour antigen (NY-ESO-1, a cancer-testis-specific antigen which is normally expressed in testicular germ cells) and IMM60, invariant natural killer T cell (iNKT) activator to induce anti-tumour responses in New York oesophageal squamous cell carcinoma-1-positive patients [232]. The clinical trial is still going on with higher CD8 + T cell responses against this epitope. The increased activation of human iNKT cells by IMM60 is expected from 15 patients under trial in this study. In a pilot study with 40 patients with single-group assignment, nanochip technology (immuno-tethered lipoplex nanoparticle [ILN] biochip) is being analysed in monitoring the treatment response and in detecting the relapse in patients with diffuse large B-cell lymphoma [233]. The study is expected to find genetic markers for diffuse large B-cell lymphoma. Hafnium oxide-containing NPs, NBTXR3, were used in phase 1 clinical trial for pancreatic cancer cells through radiation therapy [234], and also against head and neck squamous cell cancer (HNSCC) with improved efficacy of radiation therapy [235, 236]. Treatment with NBTXR3 was also found to be safe and tolerable for HNSCC patients where 11 out of 12 patients showed high clinical success. Patients in this study were given either a single-arterial injection or single intratumour injection of NBTXR3 on first day followed radiation therapy 2 h later that continued up to 7 weeks. Radiotherapy was persisted with all patients unless their tumour was shrunken to ≤ 50% of the baseline size. In this phase 1 trial, no serious adverse/side effects or dose-limiting toxicities (DLTs) were observed, which allowed all the patients to persist with NBTXR3 treatment as planned. The high success of NBTXR3 allowed the FDA to grant fast track approval to NBTXR3 in 2019 for HNSCC patients who are ineligible for platinum-based chemotherapy.

Albumin-bound NP with rapamycin (known as nab-rapamycin) in combination with pazopanib hydrochloride is in the clinical trial (phase1/2 and 57 patients) which is expected to halt the tumour growth by blocking the growth of enzymes that target advanced non-adipocytic soft tissue, solid tumours, glioma, and glioblastoma [237]. In a parallel study to target solid tumours, nab-rapamycin is used with temozolomide, and irinotecan as a combinational therapy to measure the efficacy towards the solid tumours of 33 paediatric patients in a phase 1 study [238]. Paclitaxel in the same form (Nab-paclitaxel) is being used to improve therapeutic efficacy by combining with gemcitabine + ascorbic acid instead of alone gemcitabine for the treatment of pancreatic cancer [239]. The phase 1 study has indicated the MTD of high-dose ascorbic acid with triple therapy of protein-bound NP paclitaxel + cisplatin + gemcitabine (NABPLAGEM) in metastatic pancreatic cancer patients with advanced stage IV. Magnetic NPs coated with EpCAM and CD52 antibodies are used as ligands to bind with targets of prostate, colon, lung, or pancreatic cancer or leukaemia [240]. A cetuximab-loaded NP generated from ethyl cellulose is a pH-responsive nanomaterial in which the antibody is targeted for colorectal cancer cells (33 patients with phase1 randomized study) and is stimulated to release only at pH > 6.8, while at pH < 6.8 the drug remains tightly attached to the NPs indicating a TME-based delivery.

## Challenges of nanomedicines for cancer therapy

### Regulatory approvals and barriers

There are problems with the regulations of nanomedicines. There is no unified definition or classification of nanomedicines/nanoformulations/nanomaterials with variable analytical methods for each nanomaterial. The Nanomedicines ExpertGroup (formed by the EMA) has created multiple initiatives, like the Nanomedicine Characterization Laboratory (NCL) and the Regulatory Science Framework for nanomaterial/biomaterial-based Medical Products and Devices known as REFINE under the project http://refine-nanomed.eu/. The purpose is to form guidelines and definitions for the proper regulation of nanomaterials and provide an insight on preclinical experimental methods of using them [241]. The FDA first published draft guidance on nanomaterial-based products, in 2017 [242]. The Nanotechnology Task Force and the Nanotechnology Interest Group which are comprised of experts from many regulatory agencies are formed by the FDA to control the issue of nanotechnology regulation worldwide. However, a clear descriptive guideline is still missing. Nanomedicines in Japan are controlled and regulated by the Ministry of Health, Labour and Welfare (MHLW) and Medical Devices Agency (PMDA). In 2016, the MHLW prepared a guideline for the development of liposomal drugs which was accepted by many other regulatory agencies. In Canada, the Canadian Institutes of Health Research (CIHR) regulates the nanomedicine production guideline which is very similar to the FDA guidelines. In 2019, the first guidelines for nanomedicine regulation were published by the Indian government [243]. There are few complications or lack of unified decisions on regulations of non-material. For example, there is no standard, universally accepted definition or classification of nanomedicines which can vary for a single drug-loaded nanomaterial drastically with respect to the size, shape, charge, etc. It is very difficult to regulate the pharmacokinetic (PK) property of a nanomaterial. Generally for nanomaterial, the PK data are analysed for the entrapped/coated drug although it is associated with a carrier (metallic NPs, CNTs, polymer, hydrogel, etc.) for which the PK profile may be complexly different but that is rarely addressed. Further, there are no clear guidelines for stability studies where based on physiochemical properties the same drug with different nanocarriers may come with different stability study protocol. Another problem is the confusing definition between medicine and medical devices for the same nanomaterial based on his preferred applications in different parts of the world [244]. To create better nanotechnology value chains and the global commercial eco-system the Nanotechnology Industries Association (NIA) was created in 2005 (https://nanotechia.org/about) with its head office in Brussels, Belgium. The NIA supports all its members in 3 key areas which include scientific advancement, business development through potential clients, and regulatory support. Under the regulatory support, the members were trained with a regulatory monitoring database and a global regulatory working group. The formation of the nanotechnology world association (https://www.nanotechnologyworld.org/about) is a perfect ensemble of collaboration of academia and industry for nanomedicine-based product development through concise regulatory and Organisation for Economic Co-operation and Development (OECD) guidelines.

### Production and scalability

The most challenging step in the product development for nanomedicines is the transition from small batch scale in laboratory to large industrial volumes since minor variations in physiochemical properties (size, shape, charge, etc.) can drastically change the PKPD properties of nanomaterials [245]. The synthesis of nanomedicines is generally done by two approaches—“top-down” or “bottom-up”. Top-down methods start with larger (macroscopic) starting materials, whereas bottom-up starts with constructing complex materials from simpler atomic/molecular constituents. Examples of bottom-up approaches include sol–gel processing, aerosol-based, precipitation, microfluidics, chemical vapour deposition, nanoemulsions, and chemical-based conjugation& crosslinking, etc. The manufacture of liposomal formulations (e.g. Doxil®) follows bottom-up approaches. PNPs at the laboratory scale can be obtained by solvent evaporation, emulsification, salting-out, solvent displacement, etc.; however, the solvent displacement method gives poor industrial-scale output [246]. Further, supercritical precipitation, electro-spraying, or spray-drying methods have shown success for nanomaterial production. Therefore, bioconjugation approaches and self-assembly strategies are required to achieve ADCs on a large scale [247]. In 2005, an organization called ETPN (European Technology Platform on Nanomedicine, a collaboration between EC and pharmaceutical Industry) was created which offers free-of-cost services related to nanomedicine product/clinical development through the organization’s translation advisory boards and GMP manufacturing guidelines to all members including academic laboratories, entrepreneurs and small & big industries [247]. The FDA and ICH (International Council for Harmonization) introduced the concept known as Quality-by-Design (QbD) which supports the new chemical entity (NCEs) identification, analysis, quality, and safety which is rigorously applied for nanomedicine-based products.

### Toxicity and safety

Apart from synthesis, there is a big challenge for nanomaterials to show targeted effects with reduced toxicity. It has been observed that most of the nanoformulation that has moved from preclinical to phase 1 trial are mostly liposomal and polymeric NPs. Although metallic NPs (GNP, SNP, ZnO, INPs) are more often studied in the laboratory, their progress from preclinical to clinical trials is virtually rare even though there are many reports of biogenic/green synthesis of metallic NPs with significantly reduced toxicity in vivo against animal models.

The metabolic fate of metallic NPs is considered a problem since it downregulates the Human Cytochrome P450 Enzymes. Over the years it was observed that research work and publications are more with GNPs and INPs although ZnO NP is found to be more tolerable in terms of toxicity [248]. The long-term effect of ZnO NPs was determined on mice model using doses as high as 500 mg/kg and 5000 mg/kg. It was observed that 500 mg/Kg of ZnO NP causes no toxicity even after 35 weeks, whereas 5000 mg/kg doses cause a significant reduction in body weight within 4–15 weeks with increased uptake in the liver and > 30-fold over-expression of Zn metabolizing enzymes [249]. Studies on different AgNPs with variable sizes showed that the AgNPs accumulate in the liver primarily irrespective of the delivery systems which are intravenous, oral, and subcutaneous or inhalations [250]. This kind of data from the animal model creates doubt for planning a clinical trial since AgNP-based delivery may not be tumour and organ-specific. The PEGylated GNPs, which have longer circulation time than normal GNPs, showed that increasing doses of 13 nm GNPs on rabbits does not reflect any significant changes in elimination half-life (T1/2), the volume of distribution (Vd), or mean residence time (MRT) in the animal body [251]. More importantly, with the increased size of GNPs the plasma concentration of GNPs drastically (> 100 fold for 4 nm vs. 100 nm GNP) decreases with similar doses. The presence of a positive charge on the GNPs (10 nm) compared to a neutral or negative charge indicated a > tenfold increased C_max_ for the positively charged GNPs [252]. Therefore size, shape, surface charge, and coating drastically change the pharmacokinetic properties of the metallic NPs creating doubt for their selection in clinical trials. Due to their inherent fluorescence properties QDs can be used in real-time fluorescent imaging of cancer cells by detecting specific cancer markers, such as HER2, PSA, folic acid, and CD44, etc., by attachment of peptide, monoclonal antibody or receptor-specific ligand to the QDs. However, their long-term use is questionable. For example, the QDs generated from periodic table elements of II-VI (like CdSe where Cd is from table II and Se is from table VI) liberate toxic ionized heavy metals [253]. Therefore, they always have to be protected, by a shell of PEG or other biomaterial during delivery. Therefore, CdSe-based QDs application for bioimaging of cancer may be problematic. For CNTs, it was evident that MWNTs are more toxic than SWNTs with similar size, whereas with increasing size the toxicity increases for both. The general mechanism of uptake of most GQDs, CNTs is through caveolin-mediated endocytosis which, in general, causes ROS-mediated apoptosis/necrosis only at higher doses. To date, the most common UCNPs are from ytterbium and thulium-doped sodium yttrium fluoride (NaYF4: Yb, Tm) which are highly luminescent. These UCNPs are more biocompatible and less cytotoxic as measured from *C. elegance* models [253, 254].

The growth of the human gut bacterium *Lactobacillus casei*, a probiotic, is reduced in the presence of SNPs and GNPs when tested in vitro against simulated intestinal fluids indicating the possible bad effect of the metallic NPs [255]. However, a recent study suggested that there is a common fate after metabolism for GNPs, which was analysed inside primary fibroblasts for 6 months, where < 25 nm of GNPs is degraded by NADPH-oxidase to < 3 nm of gold clusters which eventually excreted out of the body causing limited toxicity [256]. These kinds of study results indicate a future hope for metallic NP-based drug delivery with possible clinical success. AurImmune, a GNP conjugated (~ 27 nm) with thiolated PEG and tumour necrosis factors alpha (TNFs-α), progressed in phase III clinical trials for the treatment of advanced cancers. A Phase I study of CYT-6091 in 2005 which enrolled 29 patients with solid cancers showed dramatic improvement in tumour targeting with reduced toxicity when PEG-thiol was used with GNP-TNF- α [257]. AuroShell, a thin layer of gold that is coated on silica particles, is another example of metallic NPs that went through clinical trials with successful use in photothermal therapy or along with radiation or standard cancer chemotherapy [257].

### Failure and discontinuation of nanomedicine trials and drugs

Since the approval of Doxil in 1995, till date only a few nanomedicine-based drugs have been approved for the treatment of cancer. Among them, NBTXR3 (hafnium oxide NP), which is used for locally advanced soft-tissue sarcoma, is the latest one which was approved by the EU in 2019. The current clinical trial statistics indicate that maximum cancer nanomedicine drugs failed to proceed further from Phase I trials. An analysis of the clinical trial data in 2019 indicated that the success rates of phase 1, 2, and 3 trials of nanomedicines significantly decreased from 94 to 48% to 14%. In 2016, the clinical trial of MRX34 (a liposomal formulation of miRNA-34a for the treatment of cancer) was stopped in phase I as > 20% of patients faced severe immune-related adverse events [258]. The Phase I study of MM-310 (liposomal formulation of docetaxel) was terminated in 2019 due to the side effect of cumulative peripheral neuropathy. Even after succeeding in phase II trials, the phase III trial of BIND-104 (docetaxel NP) failed due to the improper payload which affected the pharmacokinetics. Apart from failure in clinical trials there are examples of drugs which are with-drawn/replaced from the market due to various reasons. The anticancer drug DepoCyt (a liposomal formulation of Cytarabine) was approved by the FDA in 1999 but was withdrawn from the market in 2017 due to a constant technical problem with large-scale manufacturing of the drug. Feridex (FDA approved in 1996), a colloidal superparamagnetic iron oxide NP associated with dextran, was used for MRI using intravenous application, but it has been withdrawn from the market now. Although the reason for discontinuation is never announced publicly, it is likely that complex manufacturing process of nanomedicine is a major hurdle apart from the failure in the clinical trials for drug development. In 2012, due to manufacturing problems, the production of Doxil was stopped and temporarily replaced by doxorubicin hydrochloride liposome injection (Lipodox) by Sun Pharma to maintain the demand.

### Problems with protein corona

The protein corona (PC) on the surface of NP creates an interface between macromolecules and NPs, thereby governing the PKPD properties of the functionalized drug. Further, the PC is generally formed by the opsonins from serums which are easily internalized by the mononuclear phagocyte system (MPS), thereby reducing the bioavailability of the NP-drug conjugate [259]. Generally, the thickness of the PC is measured by TEM and/or SEM, while the composition of the proteins is analysed by mass spectroscopy (MS)-based proteomics studies or SDS-PAGE analysis for quantitative and qualitative purposes, respectively [260]. Walkey et al. summarized the data from 26 studies with 63 nanomaterials to identify the number and abundance of proteins in the PC. The study reported that there are 125 plasma proteins of which 21 are abundant among which the proteins like albumin, immunoglobulins, apolipoprotein, fibrinogen, and complement are more abundant [261]. Peng et al., did an interesting study where the association of enzymes of the gastrointestinal tract (GIT) with cationic NPs (CNPs) was evaluated for the CNP uptake on Caco-2 cells [262]. The protein pepsin which is present in simulated gastric fluid (SGF) and pancreatin which is present in simulated intestinal fluid (SIF) are used for PC formation on CNPs. It was observed that pancreatin adsorption kinetics on CNPs is much higher with much reduced cellular uptake. Zhang et al. gave an overview of the PC formation with GIT proteins and their role in NP oral delivery. One important observation is that there is almost a linear increase of PC deposition on the NPs with the increasing size of NP ranging from 3, 10 & 100 nm. [263]. The size of AgNP was found to be > 900 nm in SGF of pH 1.2 compared to < 250 nm in pH 6.2 of SIF due to the formation of PC. The in vivo fate of different NPs concerning their PC formation was analysed against different dissolution mediums composed of SGF, SIF, and sodium carbonate/bicarbonate-based buffer with or without various enzymes like amylase, pepsin, and lipase [264]. Apart from changing the PKPD properties, the PC can change or reduce the cytotoxicity of the delivered NP. For example, when gold nanorods were coated with keratin, instead of cetyl trimethyl ammonium bromide (CTAB), the cytotoxicity is reduced [265]. Liu et al. discussed the PC formation of different types of GNPs with human serum proteins, IgG, complement factors, etc. [266]. GNP molecule bound to IgG was found to reduce the immunological functions of the complement system due to the altered structure of the antibody on the NP surface. Depending on the presence of proteins in the corona the cellular uptake can be increased or decreased. Delivery of selenium NPs against MDA-MB-231 cell lines with CTAB or other non-ionic surfactants showed reduced cellular uptake due to human serum albumin (HSA) and IgG-based corona formation [267]. However, if the PC contains high transferrin on positively charged CTABs, the cellular uptake of selenium NPs against cancer cells is increased. In an interesting study, the formation of protein PC was studied against cancer cells with citrate-, SiO_2_-, and phospholipid micelle-coated rare-earth doped NPs (RENPs). The accumulation of citrate-RENPs was more for MDA-MB-231 cells than the MCF-7 cell line [268]. The macropinocytosis-based uptake in both breast cancer cell lines was increased due to proteins present in the PC of SiO_2_-RENPs. Polymeric NPs generally contain fewer amounts of PC and with 100 nm of PLGA or PLGA-PEG NP, the PC formation was found to be independent of polymer composition. In general, the formation of PC affects the drug-targeting since the functionalized drug is masked by PC. However, the toxicity and uptake of PC-forming NP may vary based on the type of proteins that are present in PC.

## Future of nanomedicines for cancer therapy

Overcoming drug resistance against cancer is probably the biggest challenge apart from the problems associated with the side effects of chemotherapy. The side effect generally happens due to off-target hits of chemotherapeutics against the tumour cell forcing longer treatment durations and, thereby, generating drug resistance. The surface modification of NPs with liposomes, biodegradable polymers, and tumour-targeted molecules/antigens/ ligands allowed them to evade the uptake of NPs by the mononuclear phagocytic system (MPS), thereby increasing the residence time in circulation, and increased uptake through the vasculature on tumour targets. The major receptors for cancer target are CD44 (a transmembrane hyaluronic acid receptor involved in cell adhesion, migration, metastasis, and drug resistance), CD90 (a glycosylphosphatidylinositol-anchored membrane glycoprotein involved in tumourigenicity and metastasis), CD133 (a target antigen for acute myeloid leukaemia), and aldehyde dehydrogenase (ALDH, responsible for detoxification of anti-cancer drugs by converting aldehydes to carboxylic acids). Using nanoformulation-based delivery either active targeting or passive targeting is achieved [269]. In passive targeting enhanced permeability and retention (EPR) is observed, while in active targeting, NPs (conjugated with antibodies, peptides, aptamers, and other small molecules) are used for targeting specific tumour sites with specific receptors. During active targeting stimuli-responsive NPs can deliver the payload in a controlled slow release like pH-responsive (in TME), photo-responsive (during PDT), and fluorescence-responsive (during imaging) drug delivery. The FDA approval of Abraxane, a PrNP, and clinical trial of other PrNPs gives specific advantages since macromolecular protein structure allows high drug load, amino acid side chains enable multiple chemical modifications, and protein-based targeting gives more biocompatibility and circulation half life of the loaded drug. Modern-day advancement of GQD-, CNT-, and UCNP-based drug delivery comes with enormous photophysical properties enabling PDT, bioimaging, and payload delivery by a sub-nanometre vehicle. These phenomena give multiple advantages for NP-based drug delivery where a single NP-based vehicle can be used for drug delivery and/or bioimaging/biosensor, thereby tracking the payload for a longer period inside the tumour sites. Drug delivery using NPs reduces toxicity in healthy cells, provides drug stability, and improves solubility, drug loading capacity, and half-life. Contrary to conventional chemical cancer treatment methods, NP-based cancer treatment provides better biocompatibility, specificity, controlled drug release, less cytotoxicity, prolonged half-life, and high loading capacity of drugs [269].

## Conclusion

The advancement of CAR- T cell therapy and ADCs have made significant progress in the treatment of various cancers and, even, in personalized medicines. However, the high cost and need for high-end technology for the production of CAR- T cells and ADCs limit their broader use. Therefore, to date, chemotherapy is the major option even though there are side effects and increased reports of drug resistance. A recent trend has shown that virtually all major chemotherapeutics (5-FA, Cisplatin, Paclitaxel, DOX, etc.) were mostly tried in a nanoformulation, at first, rather than making a derivative from those actives for drug delivery [270–272]. This is purely because a significantly increased efficacy was observed both, in vitro and in vivo, for these nanoformulations which are much easier and cheaper to deliver in a nanoformulation rather than a chemically modified derivative of those actives. Decreased toxicity, targeted drug release, enhanced PKPD properties and dual advantages of therapeutic delivery & imaging attract nanoscale platform not only for cancers but, nowadays, and even for neglected tropical diseases [273, 274]. Nanomedicine can be used to improve more sensitive and specific cancer biomarker detection which may, in turn, provide better prevention than care. Another biggest advantage of NP-based delivery is its stimuli-responsive (pH, temperature, photochemical, radiosensitization, redox-active, magnetic, etc.) properties which make them versatile and specific for targeted delivery. In cancer, the observed high average success rate of 94% of studies in phase I clinical trials drops significantly to < 50% in phase II and < 15% in phase III trials. With such a low success rate it is really difficult to proceed with new chemical entities (NCEs), whereas the NP-based formulation provides a quick bypass since liposomal and polymeric NPs generally overcome the barrier of Phase 1 trials. In the nanoscale delivery still, there is a general trend of preference for liposomal formulation (liposomal >  > Polymeric > protein > metallic NP) over other formulations. However advanced clinical trials, FDA approval, and successful application of more polymeric NPs (Oncaspar, Eligard, Zinostatin, Lipusu, etc.), few protein NPs (Abraxane, Ontak, Kadcyla, etc.) and GNP NP (Aurimmune or CYT-6091) in the last two decades have created a better hope for wide range of NP-based delivery for treatment of cancers [275]. Based on a current report there are currently > 485 clinical trials for nanomedicines in different phases for treatments of various cancers with a hope that nanotechnology-based platforms will become a better alternative than standard chemotherapy [276]. It is quite obvious that unless there is a successful discovery of vaccines for cancers, the chemotherapy and subsequent nanoscale delivery of chemotherapeutics will remain a mainstay for cancer treatment.

## Data Availability

Data sharing is not applicable to this article as no datasets were generated or analysed during the current study.

## Abbreviations

QDs: Quantum dots
CNTs: Carbon nanotubes
SWCNTs: Single-wall carbon nanotubes
MWCNTs: Multi-wall carbon nanotubes
ROS: Reactive oxygen species
PEG: Polyethylene glycol
PEI: Poly(ethyleneimine)
PLGA: Poly(lactide-co-glycolide)
PLA: Poly(D,l-lactide)
PLG: Poly(D,L-glycolide)
EPR: Enhanced permeability and retention
ABCs: Amphiphilic block copolymers
PNPs: Polymeric nanoparticles
PrNP: Protein nanoparticles
GNPs/AuNPs: Gold nanoparticles
MPS: Mononuclear phagocyte system
GO: Graphene oxide
SPION: Superparamagnetic iron oxide nanoparticle
MRI: Magnetic resonance imaging
INPs: Iron oxide magnetic nanoparticles
PDT: Photodynamic therapy
PS: Photosensitizers
MTD: Maximum tolerated dose
TME: Tumour micro-environment
NP: Nanoparticle
PrNP: Protein nanoparticles
UCNP: Upconverting nanoparticles
TAA: Tumour-associated antigen
HA: Hyaluronic acid
PD-L1: Programmed death Ligand 1
PD-1: Programmed death 1
DC: Dendritic cells
TLR: Toll-like receptor
HNSCC: Head and neck squamous cell cancer
